# Approach of a small protein to the biomimetic bis-(μ-oxo) dicopper active-site installed in MOF-808 pores with restricted access perturbs substrate selectivity of oxidase nanozyme†

**DOI:** 10.1039/d4sc02136c

**Published:** 2024-06-10

**Authors:** Rasmi V. Morajkar, Adarsh P. Fatrekar, Amit A. Vernekar

**Affiliations:** a Inorganic and Physical Chemistry Laboratory, CSIR-Central Leather Research Institute Chennai 600020 Tamil Nadu India amitvernekar@clri.res.in; b Academy of Scientific and Innovative Research (AcSIR) Ghaziabad-201002 India

## Abstract

Advances in nanozymes have taken shape over the past few years in several domains. However, persisting challenging limitations of selectivity, specificity, and efficiency necessitate careful attention to aid in the development of next-generation artificial enzymes. Despite nanozymes having significant therapeutic and biotechnological prospects, the multienzyme mimetic activities can compromise their intended applications. Furthermore, the lack of substrate selectivity can hamper crucial biological pathways. While working on addressing the challenges of nanozymes, in this work, we aim to highlight the interplay between the substrates and bis-(μ-oxo) dicopper active site-installed MOF-808 for selectively mimicking oxidase. This oxidase mimetic with a small pore-aperture (1.4 nm), similar to the opening of enzyme binding pockets, projects a tight control over the dynamics and the reactivity of substrates, making it distinct from the general oxidase nanozymes. Interestingly, the design and the well-regulated activity of this nanozyme effectively thwart DNA from approaching the active site, thereby preventing its oxidative damage. Crucially, we also show that despite these merits, the oxidase selectivity is compromised by small proteins such as cytochrome *c* (Cyt *c*), having dimensions larger than the pore aperture of MOF-808. This reaction lucidly produces water molecules as a result of four electron transfer to an oxygen molecule. Such unintended side reactivities warrant special attention as they can perturb redox processes and several cellular energy pathways. Through this study, we provide a close look at designing next-generation artificial enzymes that can address the complex challenges for their utility in advanced applications.

## Abstract 2

Development of substrate-selective nanozyme requires careful attention. We show that proteins with dimensions larger than the pore aperture of MOF-808, installed with a bis-(μ-oxo) dicopper active site in its adamantane pore, can compromise the substrate selectivity of oxidase nanozyme.

## Graphical abstract

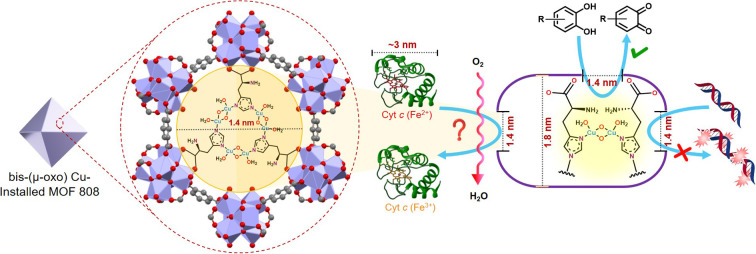

## Introduction

Natural enzymes are highly selective, capable of choosing a single substrate amongst the closely related substrates, and are very specific towards a given reaction. This specificity arises from the enzymes' three-dimensional (3D) structure within its active site, which involves a complex interplay regulated by the amino acid residue projected in binding pockets.^1–3^ Nevertheless, achieving this level of sophistication in synthetic constructs such as small molecules, supramolecular systems, and nanomaterials remains a formidable challenge. In the frame of catalytic nanomaterials, nanozymes have gained immense interest, mainly due to their potential catalytic features, low cost, and improved stability.^4,5^ The term ‘nanozyme’ first appeared in 2004 for the immobilized triazacyclononane/Zn^2+^ on Au nanoparticles used for transphosphorylation activity actually originating from the Zn complex.^6^ In 2007, the report on magnetic nanoparticles (Fe_3_O_4_) appeared demonstrating its intrinsic peroxidase-like activity for the first time.^7^ The field has rapidly expanded since then, encompassing diverse nanomaterials and single-atom nanozymes.^8–10^ However, the field is still in its infancy due to several critical limitations, including non-biocompatibility, low catalytic activity at physiological pH, and lack of specificity. Despite numerous efforts to synthesize nanozymes with diverse morphologies, compositions, and functions, they still lack selectivity and function as multienzyme mimetics.^11–14^ For instance, nanozymes displaying antagonistic catalytic properties, particularly peroxidase and antioxidant enzyme-like activities, exhibit reactive oxygen species (ROS) generation and elimination, rendering them unsuitable for intended biological applications.^15–18^ This catalytic versatility can result in unintended side effects when used in therapeutics, as it can evoke abnormal interactions with several biomolecules, thereby upregulating or downregulating various biological pathways, leading to complications.^19,20^ The underlying reason could be the well-exposed active site for catalysis, which can readily interact with various substrates, compromising the selectivity of nanozymes. The utilization of molecular imprinting strategy in artificial enzyme fabrication has been reported as a unique approach that possesses several advantages in terms of substrate binding affinity.^21,22^ However, it poses certain limitations such as structural rigidity and poor mass transfer.^23^ Therefore, it is imperative to explore alternative strategies that can overcome these limitations and enable the design of artificial enzymes with enhanced performance and specificity. In this regard, understanding the critical details governing the enzymatic properties is essential in achieving catalysts-by-design,^24,25^ rather than general screening of nanomaterials for enzyme-like activities.

Recently, 3D-metal–organic frameworks (MOFs) have emerged as a notable solution, providing a flexible approach to designing artificial enzymes that incorporate several structural features. These features include nanoconfined active sites and a unique diffusion channel environment to impart selectivity and provide a secondary coordination sphere-like microenvironment.^26,27^ MOFs with interconnected pores closely resemble the substrate channels in enzymes. Recently, MOF-818 was identified for exhibiting specific catechol oxidase-like activity without the peroxidase-like activity.^28^ Also, Wang *et al.*^29^ have reported an amino acid-treated copper MOF that selectively catalyzes ascorbate, useful for targeted ascorbate sensing in sweat.

Among the various reported MOFs, MOF-808 is deemed to be the most suitable owing to its exceptional structural features and high stability.^30^ Notably, Yaghi and co-workers reported a remarkable MOF-808 construct with biologically relevant ligands installed in its pore (bis-(μ-oxo) dicopper) which has shown catalytic activity for selective methane oxidation.^31^ Furthermore, Zhu and co-workers^32^ have explored this construct for catechol oxidase-like activity, which demonstrated recognition and oxidation of chiral catechol substrates by incorporating a chiral histidine ligand. These findings are highly promising in the domain of designing enzyme mimics with selectivity.

While several reports highlight complex designs, efficiency, specificity, novel applications and mechanisms of nanozymes,^33–49^ the lack of studies on side reactivities that can complicate their mode of action projects a huge gap in this field. We have also been working on the development and addressing challenges of nanozymes and catalysts with enzyme-like functions.^50–55^ In this study, we aim to showcase that although the strategies employing pore-engineering of MOFs are essential for regulating selective catalysis of small molecules over complex biomolecules, the activity can still be compromised by small proteins. This could trigger inappropriate activities during the intended function of nanozymes, thus affecting the outcome. Herein, we systematically show that biomimetic bis-(μ-oxo) dicopper active sites, closely resembling the dinuclear Cu center from the active site of catechol oxidase and tyrosinase,^56^ installed in the pores of MOF-808 (MOF-808-His-Cu) exhibit remarkable substrate-specific oxidase activity towards catechols. As the active site is present inside the adamantane pore with an aperture diameter of 1.4 nm, it serves as an interesting strategy to control the approach of substrates, their dynamics and reactivity. This construct is a selective oxidase mimic that draws a line from other oxidases, peroxidases, catalase and glutathione peroxidase (GPx) nanozymes. Oxidase mimics, especially, metal complexes and nanomaterials having open structured active sites can interact with cellular organelles and potentially damage them by catalytically generating ROS.^57–60^ Interestingly, in MOF-808-His-Cu, the oxidase-like active sites are under the tight control of pore dimensions (aperture diameter of 1.4 nm) of MOF-808, and therefore, are difficult to access by complex biomolecules like DNA, preventing their ROS-mediated damage. Several oxidase nanozymes, including Cu-containing counterparts, have a potential role in biomedical applications and redox functions.^10,57,59,60^ However, it is important to note that most of these nanozymes can non-selectively interact with biomolecules, thus they can potentially interfere with various cellular redox processes, electron transfer and energy pathways. Furthermore, such reactivities may lead to a negative impact on the other vital biological processes. Thus, it is highly essential to probe the side reactivities that could impact its clinical translation. In this regard, we thought it worthwhile to check the interactions of a small protein involved in the electron transport chain and energy metabolism, cytochrome *c* (Cyt *c*), with MOF-808-His-Cu oxidase nanozyme. While it is understood that the aperture of 1.4 nm helps this construct efficiently keep complex biomolecules away from approaching the active site, we systematically show that proteins such as Cyt *c* are capable of accessing the active site, compromising substrate selectivity. The approach of Cyt *c* to MOF-808-His-Cu leads to a redox reaction similar to Cyt *c* oxidase (COX), majorly leading to four electron oxygen reduction to water. Thus, selectivity in such biomimetic systems is compromised, highlighting a critical look at designing nanozymes with selectivity for clinical applications. While research on imparting selectivity to nanozymes is at the forefront of the domain, a thorough investigation is needed to address the challenges posed by newer solutions. Our research findings highlight the crucial factors to be considered when designing next-generation enzyme mimetics with high efficiency, substrate and enzyme mimetic selectivity.

## Results and discussion

### Synthesis and characterization of MOF-808-His-Cu

**Fig. 1:**
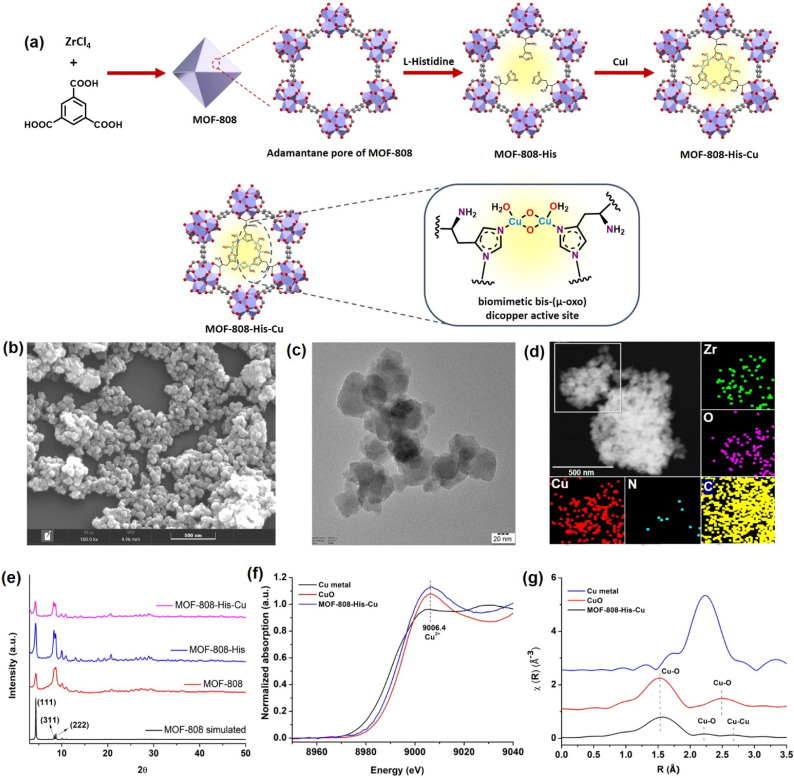
(a) Schematic illustration of the synthesis of MOF-808-His-Cu with the biomimetic bis-(μ-oxo) dicopper active site installed inside the adamantane pore and its expanded view. (b) SEM and (c) TEM images of MOF-808-His-Cu. (d) Elemental mapping highlighting the spatial distribution of Zr (green), O (pink), C (yellow), N (blue), and Cu (red) in MOF-808-His-Cu. (e) PXRD spectra of synthesized materials compared with the simulated MOF-808 crystal structure. (f) Normalized Cu K-edge XANES spectra of MOF-808-His-Cu, along with the reference compounds (Cu metal and CuO). (g) *k*^2^-weighted Cu-EXAFS *χ*(*R*) spectra of MOF-808-His-Cu, along with the reference compounds.

The nanoscale MOF-808-His-Cu was fabricated by following the reported procedure with slight modifications with respect to the size of the MOF.^31,61^ We intended to have a size of the MOF-808 in dimensions below 100 nm because of its relevance in biological applications, especially in cell-based studies.^62^ The microcrystalline structure of MOF-808 is composed of the secondary basic units (SBUs) Zr_6_O_4_(OH)_4_(COO)_6_(HCOO)_6_ connected by a 1,3,5-benzenetricarboxylate (BTC) linker.^63^ This MOF has two different interconnected pores: the tetrahedral pore (having an internal diameter of 0.48 nm and aperture diameter of 0.12 nm) and the larger adamantane pore (having an internal diameter of 1.8 nm with an aperture diameter of 1.4 nm).^63,64^ To incorporate l-histidine in the adamantane pores, a post-synthetic modification was performed by binding the carboxylate groups of l-histidine to Zr(iv) in nodes.^65^ This functionalizes the amino groups to the interior of the pores, serving as the coordination site for Cu metal ions inside the pores (Fig. 1a and S1†). This fine-tuning and decorating of the pores can serve as reaction pockets analogous to the enzyme binding pockets. As shown by the scanning electron microscopy (SEM) of MOF-808-His-Cu (Fig. 1b), it can be observed that the morphology has been retained after modification with l-histidine and Cu^2+^ ions (Fig. S2a and b†). From the high-resolution transmission electron microscopy (HRTEM) image (Fig. 1c, S2c and d†), it can be observed that the particle size of the MOF-808-His-Cu is in the range of 80–100 nm. The energy-dispersive spectroscopy (EDS) mapping images suggest the uniform distribution of Zr, Cu, O, C, and N elements which further confirms the successful incorporation of l-histidine and Cu^2+^ ions in the MOF-808 framework (Fig. 1d). The powder X-ray diffraction (PXRD) analysis revealed that the phase purity and crystallinity of the synthesized MOF-808 were well preserved upon post-functionalization with l-histidine and metalation with Cu^2+^ ions (Fig. 1e).^31,63^ The porosity of the samples was confirmed from the N_2_ adsorption–desorption isotherm recorded at 77 K and the Brunauer–Emmett–Teller (BET) surface areas of MOF-808, MOF-808-His, and MOF-808-His-Cu were found to be 868, 752, and 482 m^2^ g^−1^, respectively (Fig. S3†). The decrease in the surface area on functionalization can be attributed to the space occupied by the l-histidine and Cu^2+^ ions in the pores.^65^

The successful anchoring of l-histidine and the mode of binding of Cu^2+^ ions as Cu-(μ-oxo)-Cu species were observed by UV-visible diffuse reflectance spectroscopy (UV-vis DRS) (Fig. S4†).^31,66,67^ The ^1^H NMR spectra of the digested MOF samples further manifest the loading of l-histidine to reveal the degree of functionalization, *i.e.*, 3 histidine ligands per chemical formula as reported earlier (Fig. S5–S7†).^63,65^ The loaded Cu content was found to be 7.4 wt% from inductively coupled plasma optical emission spectroscopy (ICPOES). The X-ray photoelectron spectroscopy (XPS) analysis for MOF-808-His-Cu confirms the presence of Zr, C, N, O and, Cu (Fig. S8a†). The deconvoluted spectra of Zr 3d (Fig. S8b†), C 1s (Fig. S8c†), O 1s (Fig. S8d†) and N 1s (Fig. S8e†) and their binding energies are in agreement with previous reports.^68^ The high-resolution Cu 2p spectrum exhibits the characteristic peaks of Cu 2p_3/2_ (932.97 eV) and Cu 2p_1/2_ (952.81 eV) that are otherwise indicative of Cu(i) and Cu (0) (Fig. S8f†).^69,70^ However, the covalent interactions of ligands with Cu^2+^ may lead to a decrease in the Cu 2p binding energy as a result of the electron transfer from the ligand to Cu^2+^, which is well supported by previous reports.^71–73^ Furthermore, the oxidation state of Cu was confirmed by X-ray absorption near-edge structure (XANES).^74^ The Cu K-edge XANES and extended X-ray absorption fine structure (EXAFS) analysis provide further insights into the oxidation state and coordination environment of Cu. From the Cu K-edge XANES profile (Fig. 1f and S9a†), the absorption edge at about 9006.4 eV is recognized as the dipole-allowed Cu^2+^ 1s → 4p transition.^66,75^ The Fourier transform Cu K-edge EXAFS spectra in the *k*-range of (3–11) Å^−1^ and the fitting results are presented in Fig. 1g, S9b and c.† The fitted parameters are summarized in Table S1,† showing the coordination number of 2.7 assigned to the first shell of Cu–N/(O) coordination at a distance of 2.00 Å. For the Cu^…^Cu contribution, the coordination number was fixed as 1 and the distance was determined to be 2.52 Å. The results are well in agreement with the literature,^31,66^ supporting the presence of bis-(μ-oxo) dicopper moieties in the pores of MOF-808.

### Catechol oxidase activity and kinetics of MOF-808-His-Cu

**Fig. 2:**
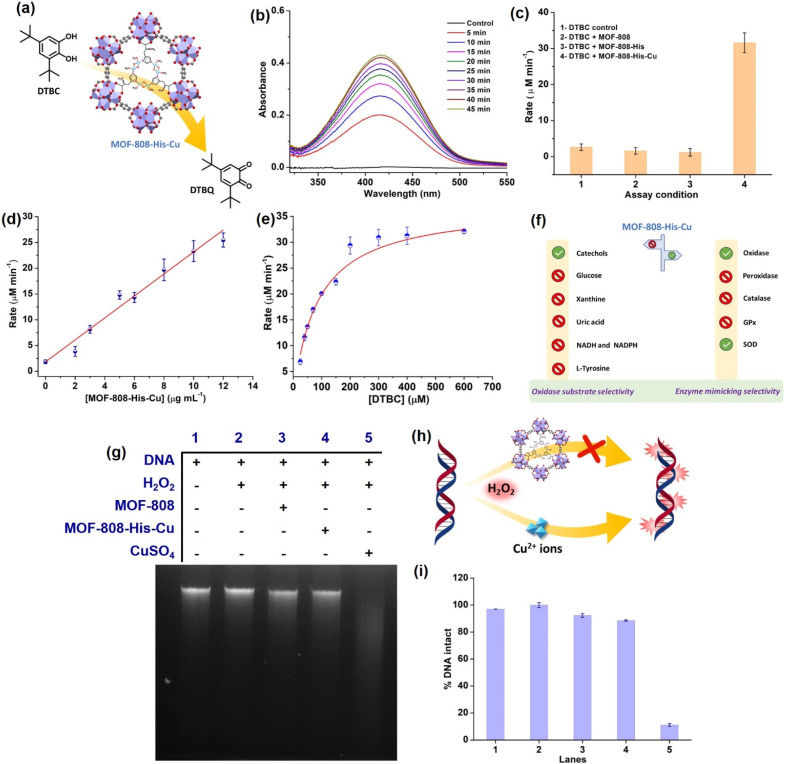
(a) Illustration depicting the catecholase-like activity of MOF-808-His-Cu. (b) Time-drive mode UV-vis absorption spectra of DTBC (200 μM) catalyzed by MOF-808-His-Cu (12 μg mL^−1^). (c) Bar diagram showing the initial rates of DTBC oxidation under different assay conditions. (d) The effect of MOF-808-His-Cu concentration on DTBC oxidation displays first-order kinetics. (e) Michaelis–Menten plot by varying DTBC concentration (30–200 μM) at a constant concentration of MOF-808-His-Cu (12 μg mL^−1^). (f) Illustrative representation showing oxidase substrate selectivity and enzyme mimicking selectivity exhibited by MOF-808-His-Cu. (g) DNA cleavage pattern of the agarose gel electrophoresis, in the presence of H_2_O_2_ (1 mM), in PB solution (pH 7.4). (h) A diagram depicting the DNA cleavage in the presence of free Cu^2+^ ions, while DNA remaining intact in the presence of MOF-808-His-Cu and H_2_O_2_ (1 mM). (i) The extent of % DNA intact plot of the corresponding lanes.

To start with, we examined the reported catechol oxidase-like activity for MOF-808-His-Cu, a biomimetic architecture that closely represents the active site of catecholase and tyrosinase enzymes.^76^ For catechol oxidase-like activity, we employed 3,5-di-*tert*-butylcatechol (DTBC) as the substrate that oxidizes to give 3,5-di-*tert*-butyl-benzoquinone (DTBQ) (Fig. 2a). As shown in , the peak intensity at 415 nm increases over time, indicating the catechol oxidase activity of MOF-808-His-Cu (12 μg mL^−1^) in phosphate buffer (PB) (pH 7.4) at room temperature (Fig. 2b). In control reactions, MOF-808 and MOF-808-His exhibit no measurable catechol oxidase-like activity, as demonstrated in Fig. 2c. The kinetic study by changing the catalyst concentration (2–12 μg mL^−1^) displayed a proportional enhancement in the activity, suggesting first-order kinetics (Fig. 2d and S10a†), which was further confirmed by plotting ln rate *vs.* ln catalyst concentration (Fig. S10b†). As depicted in Fig. 2e and S10c,† the oxidation of DTBC catalyzed by MOF-808-His-Cu resulted in a typical Michaelis–Menten kinetics. The Michaelis–Menten constant (*K*_M_) of MOF-808-His-Cu, obtained from the Lineweaver–Burk plot, is 85.07 μM, while the calculated *V*_max_ is 39.71 μM min^−1^ (Fig. S10d†). The low *K*_M_ value represents an appreciable affinity of MOF-808-His-Cu towards DTBC. The catalytic efficiency (*K*_cat_/*K*_M_) of this construct is as high as 550 M^−1^ s^−1^. A comparison of catalytic parameters of reported oxidase nanozymes for the oxidation of catechol substrates is presented in Table S2.† A comparison of these catalytic parameters suggests that MOF-808-His-Cu has an appreciable affinity as indicated above and performs catalysis with relatively higher catalytic efficiency. While the MOF-808-His-Cu shows enzyme-like activity, we tested the stability of the nanozyme by studying the leaching of ions, the effect of pH and temperature, which are crucial for its use *in vivo* and *in vitro* under physiological conditions. In the first experiment, the catalyst was incubated in 50 mM PB (7.4) for 30 minutes. After centrifugation, the supernatant was examined for DTBC oxidation activity (Fig. S11a and b†). It was observed that there was no leaching of Cu^2+^ from the MOF-808-His-Cu. In the second experiment, we tested the catechol oxidase-like activity of MOF-808-His-Cu treated under different pH conditions (pH 4, 5, 6, 7.4, 8) for 60 min. We observed that the catechol oxidation activity remained unaffected (Fig. S11c†). A slight decrease in activity is observed in the case of MOF-808-His-Cu treated under pH 4, which may be due to the loss of Cu ions at this pH. In the third experiment, we treated MOF-808-His-Cu at different temperatures (25 °C, 60 °C and 80 °C) and then tested its activity. We observed that the MOF-808-His-Cu showed exceptional temperature stability as its enzyme-like activity remained unaffected (Fig. S11d†). However, natural polyphenol oxidases are known to possess very low activity at acidic pH and denature at higher temperatures (the optimum temperature range is 40–50 °C).^77^ To investigate the catalytic mechanism, we checked the formation of H_2_O_2_ in the reaction by a standard 3,3′,5,5′-tetramethylbenzidine (TMB)– horseradish peroxidase (HRP) assay. A peak appearing at 650 nm for oxidized TMB confirms the generation of H_2_O_2_ as a result of a 2-electron reduction of O_2_ (Fig. S11e†). In this context, a catalytic cycle is proposed in Fig. S11f.† Although the oxidation of catechol by natural catechol oxidase results in the corresponding quinone and water as products,^56^ MOF-808-His-Cu produces H_2_O_2_ in this reaction.

To test whether MOF-808-His-Cu can oxidize other catechol substrates, we employed quercetin (Fig. S12a†) and (+)-catechin (Fig. S13a†) in the study. Interestingly, the construct showed significant oxidase activity, under similar conditions (Fig. S12b, c, S13b and c†). Additionally, MOF-808-His-Cu showed oxidation of ascorbic acid (AA) (unsaturated diol substrate partly similar to catechols), which was monitored by measuring the consumption of AA in UV-visible spectroscopy (Fig. S14a†). The characteristic absorption peak of AA (265 nm) decreased with time in the presence of MOF-808-His-Cu (Fig. S14b and c†). Furthermore, the final product of oxidation, dehydroascorbic acid (DHA) was probed using *o*-phenylenediammine (OPD). OPD reacts with DHA to give 3-(1,2-dihydroxyethyl)furo-[3,4-*b*] quinoxaline-1-one, resulting in intense fluorescence at 425 nm (Fig. S14d†).^78^ Thus, as reported earlier the MOF-808-His-Cu displays catechol oxidase-like activity with DTBC and other typical catechols, including quercetin and (+)-catechin.

### Evaluation of substrate selectivity of MOF-808-His-Cu

Different types of natural oxidases are known to selectively catalyze the oxidation of particular biological substrates, such as glucose, β-nicotinamide adenine dinucleotide (reduced) disodium salt (NADH), β-nicotinamide adenine dinucleotide phosphate tetrasodium salt (reduced) (NADPH), xanthine (XA) and uric acid (UA).^79–83^ However, the critical challenge that hinders the development and application of artificial enzymes and nanozymes is the lack of substrate selectivity, differing from that of natural enzymes. Oxidase nanozymes reported in the literature have demonstrated the ability to oxidize a wide range of substrates.^28,84–86^ To further explore the substrate selectivity of MOF-808-His-Cu, we evaluated its oxidase activity using the above substrates. We initially investigated whether MOF-808-His-Cu catalytically oxidizes glucose. The glucose oxidase (GO) enzyme catalyzes the oxidation of glucose, leading to the production of gluconic acid and H_2_O_2_. The H_2_O_2_ generated can be probed at pH 7.4 by HRP, which oxidizes TMB to result in a blue color (Fig. S15a†).^87^ In an enzyme cascade reaction involving glucose (1 M), 1 U mL^−1^ GO, HRP, and TMB, we observed that the catalytic oxidation of glucose resulted in an intense blue color (oxidized TMB). However, even after incubation for 60 min, MOF-808-His-Cu did not exhibit glucose oxidase-like activity (Fig. S15b†). We further tested the consumption of glucose in the absence and presence of MOF-808-His-Cu (for 60 min) using a 3,5-dinitrosalicylic acid (DNS) assay, monitoring absorbance at 540 nm for the formation of 3-amino-5-nitrosalicylic acid (Fig. S15c–e†). The amount of glucose was estimated to be the same in control and experimental reactions, confirming no consumption of glucose by MOF-808-His-Cu (Fig. S15c–e†). In another experiment, we checked whether XA can be catalytically oxidized by MOF-808-His-Cu. Xanthine oxidase (XO) catalyzes the oxidation of XA to uric acid (UA) and produces H_2_O_2_ (Fig. S16a†). This oxidation process can be assessed by probing the produced H_2_O_2_ employing HRP-coupled reactions and analyzing fluorescence read-out. One such assay is 10-acetyl-3,7-dihydroxyphenoxazine (amplex red) (AR)/HRP, which effectively probes H_2_O_2_ formation, resulting in fluorescent resorufin with emission at 583 nm.^88^ A positive control was employed in the presence of XO (33.6 mU mL^−1^) under similar reaction conditions, which resulted in an intense pink color. Conversely, no emission was observed at 583 nm for XO assay when MOF-808-His-Cu was present (Fig. S16b†). We further tested the consumption of XA in the presence of MOF-808-His-Cu (for 60 min) by directly monitoring its absorbance at 270 nm in the UV-vis spectra (Fig. S16c and d†). We observed no change in the absorbance at 270 nm, confirming no consumption of XA by MOF-808-His-Cu (Fig. S16c and d†). Urate oxidase is another copper-containing enzyme that possesses a type-2 copper binding site.^89^ In this study, to determine the oxidation of UA, we monitored the consumption of UA in the presence of MOF-808-His-Cu at pH 7.4 (Fig. S17a†). There was no change in the absorbance of UA peaks on incubation with MOF-808-His-Cu for 60 min, indicating no significant UA oxidation (Fig. S17b and c†).^90^

NADH and NADPH oxidases are another class of oxidases that are involved in the oxidation of coenzymes. Considering the vital role of NADH/NAD^+^ and NADPH/NADP^+^ as redox couples in maintaining the cellular redox potential in the signalling pathway, mitochondrial function, and numerous other biological functions,^91^ we thought it worthwhile to evaluate if MOF-808-His-Cu can catalytically oxidize NADH (Fig. S18a†) and NADPH (Fig. S19a†) by UV-vis spectroscopy. Our results indicate that there was no change in the absorbance in the UV-vis spectra of both NADH (Fig. S18b and c†) and NADPH (Fig. S19b and c†), indicating no significant oxidation in the presence of MOF-808-His-Cu.

Substrates such as l-tyrosine are oxidatively converted to dityrosine by dual oxidases (DUOX).^92^ Therefore, we introduced l-tyrosine in our reaction and assessed the formation of dityrosine in the presence of MOF-808-His-Cu (Fig. S20a†). However, no catalytic activity was noted, as we did not observe the peak at 410 nm accounting for dityrosine in the fluorescence spectra (Fig. S20b†).

In a practical application scenario, multiple substrates exist simultaneously in the biological environment. Therefore, we evaluated the catechol oxidation by MOF-808-His-Cu in the presence of multiple substrates including DTBC, glucose, XA, Ty, and, UA. We observed a remarkable selectivity in the preferential oxidation of DTBC by MOF-808-His-Cu (Fig. S21a–d†). However, the rate of oxidation was slightly decreased in comparison to DTBC oxidation catalyzed by MOF-808-His-Cu as multiple types of substrates were present simultaneously.

Among all the substrates tested for oxidase activity, only catechol-based substrates were oxidized in the presence of MOF-808-His-Cu at pH 7.4 (Fig. 2f). This suggests that the biomimetic bis-(μ-oxo) dicopper with l-histidine, which closely resembles the Type-3 copper^93^ center found in catecholase, has some influential role in selectively oxidizing catechol-based substrates.

### Evaluation of the enzyme mimicking selectivity of MOF-808-His-Cu

While enzyme mimetic activities that are complementary to each other are beneficial, antagonistic activities can compromise the intended applications. There are several studies on complementary or antagonistic multienzyme mimetic activities reported in the literature.^15–17,94,95^ The nanozymes that are composed of copper-based systems have also been shown to exhibit peroxidase, superoxide dismutase (SOD), catalase, and GPx-like activities together.^96,97^ In another study, ultra-small Cu_5.4_O nanoparticles displayed SOD-, catalase- and GPx-like activity.^13^ More recently, Geng and co-workers have described a copper-based single-atom nanozyme exhibiting superior multi-enzymatic properties, including peroxidase, oxidase, and catalase-like activity.^14^ However, we thought it worthwhile to evaluate the enzyme-mimicking selectivity of MOF-808-His-Cu.

The enzymatic activities, including oxidase, peroxidase, SOD, GPx, and catalase-like activity, were performed using a standard protocol.^98–100^ AR was utilized as a substrate to assess the oxidase and peroxidase-like activity under physiological conditions (pH 7.4).^88^ Our findings, as demonstrated in Fig. S22a, b, S23a and b,† indicate that MOF-808-His-Cu did not exhibit oxidase and peroxidase-like activity, respectively, for the AR substrate at pH 7.4. In another control reaction, HRP showed immediate oxidation of AR to resorufin in the presence of H_2_O_2_ as monitored by fluorescence spectroscopy. We further evaluated the catalase-like activity by monitoring the decrease in the absorbance of H_2_O_2_ at 240 nm using kinetics mode in UV-vis spectroscopy (Fig. S24a†). Our observation reveals that there is no change in the H_2_O_2_ absorbance in the presence of 12 μg mL^−1^ of MOF-808-His-Cu. This suggests that MOF-808-His-Cu does not display catalase-like activity (Fig. S24b†). GPx is acknowledged as another antioxidant enzyme crucial for regulating H_2_O_2_ levels by utilizing the endogenous thiol, glutathione (GSH).^101^ It catalyzes the reduction of H_2_O_2_ to H_2_O, in turn converting GSH to glutathione disulfide (GSSG). Consequently, GSSG is reduced to GSH by glutathione reductase (GR) using coenzyme NADPH (Fig. S25a†). We conducted the GR-based assay by following the decrease in NADPH concentration at 340 nm. However, MOF-808-His-Cu showed no decrease in NADPH concentration, thus revealing no GPx-like catalytic activity of the nanozyme (Fig. S25b†). Another vital antioxidant enzyme-like activity is the superoxide dismutase (SOD) activity,^102,103^ which involves the dismutation of superoxide anion radical (
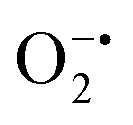
) to O_2_ and H_2_O_2_. The SOD activity was investigated by probing the 
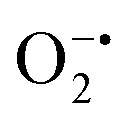
 using water-soluble WST-1 dye (2-(4-iodophenyl)-3-(4-nitrophenyl)-5(2,4-disulfophenyl)-2*H*-tetrazolium) (Fig. S26a†). The 
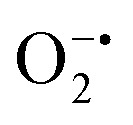
 generated from the reaction of XA and XO reduces WST-1 to formazan (yellow color), which displays a characteristic absorption peak at 440 nm.^98^ However, there was no formazan production observed for the reaction between XA and XO in the presence of MOF-808-His-Cu, indicating that this construct exhibits SOD-like activity (Fig. S26b and c†).

### Evaluation of DNA damage by side reactions of MOF-808-His-Cu

Amongst the various ROS, hydroxyl radicals are highly reactive, inducing damage to biomacromolecules (such as DNA, proteins, and lipids).^104,105^ Transition metal ions like Fe^3+^ and Cu^2+^ exhibit the Fenton reaction in the presence of H_2_O_2_, resulting from cellular processes. Moreover, H_2_O_2_ being a very small molecule can easily enter the pores of MOF-808-His-Cu and access the Cu^2+^ centers, and therefore, we tested if MOF-808-His-Cu undergoes a Fenton reaction. Addition of MOF-808-His-Cu to a solution containing H_2_O_2_ (1 mM) and terephthalic acid (TPA) reporter molecule led to the formation of hydroxy TPA, showing an emission signal at 425 nm (Fig. S27a†). This confirms that MOF-808-His-Cu produces hydroxyl radicals by the reaction with H_2_O_2_. It therefore suggests that H_2_O_2_ can easily enter the pores and access bis-(μ-oxo) dicopper sites. The Fenton reaction activity was found to be higher for CuSO_4_ as compared to MOF-808-His-Cu as seen in Fig. S27b,† indicating that the free Cu^2+^ ions interact efficiently with H_2_O_2_. To understand whether the MOF-808-His-Cu-catalyzed Fenton reaction can induce side reactions such as oxidative biomolecule damage, we tested if MOF-808-His-Cu can induce DNA damage. The *in vitro* DNA cleavage studies were performed in a vial containing 10 μg of Calf Thymus DNA (DNA, purchased from Sigma-Aldrich, product no. D3664), MOF-808-His-Cu, and H_2_O_2_ at pH 7.4 (50 mM PB), along with other controls (Fig. 2g–h). The reaction mixtures were incubated for 2 h at 25 °C and assessed by agarose gel electrophoresis. In the DNA cleavage studies performed without H_2_O_2_, we observed no change in band intensity (Fig. S27c†). While CuSO_4_ as a control degraded the DNA completely under our experimental conditions, it is interesting to note that the DNA remained completely intact even in the presence of MOF-808-His-Cu and H_2_O_2_ (lane 4) (Fig. 2i). These results are in contrast to the Fenton reaction that was probed using TPA, as discussed above. After careful analysis of the results of these experiments, we came to the conclusion that the DNA being a large molecule cannot enter the pores of MOF-808. This also further confirms that the hydroxyl radicals generated at the active site of MOF-808-His-Cu, as a result of the Fenton reaction, are prevented from cage-escape into the reaction environment. In the case of TPA, being a significantly smaller molecule than the size of the pore aperture of MOF-808, it easily enters the pores and reacts with hydroxyl radicals, forming hydroxy TPA. From these experiments, we infer that the MOF-808-His-Cu projects a tight control over substrate selectivity based on the size of molecules. This prompted us to investigate the fate of small proteins in the presence of this nanozyme.

### Probing interactions of small protein Cyt *c* with MOF-808-His-Cu

**Fig. 3:**
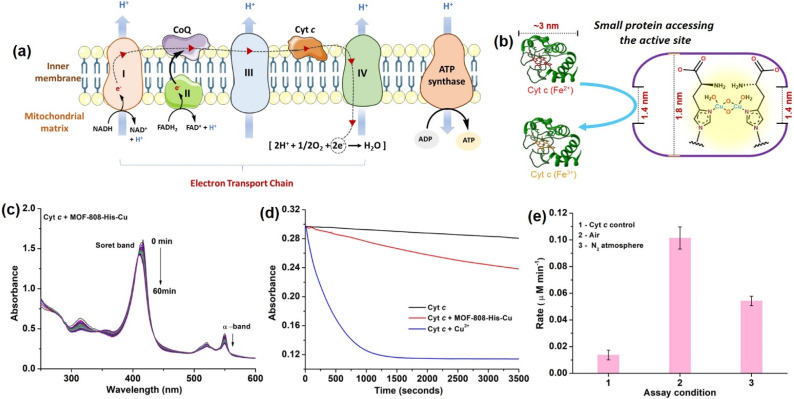
(a) Schematic diagram showing the electron transport chain in mitochondria, representing various complexes involved in the systematic transfer of electrons to Cyt *c* oxidase (complex IV), where O_2_ is reduced to H_2_O *via* a four-electron pathway. (b) Diagram depicting that Cyt *c* can access the active site of MOF-808-His-Cu despite the large dimensions relative to MOF-808 pore opening. (c) UV-vis absorption spectra of ferrous Cyt *c* (10 μM) in the presence of MOF-808-His-Cu (12 μg mL^−1^) at pH 7.4. (d) Time-dependent absorbance spectra of Cyt *c* oxidation in the presence of MOF-808-His-Cu and Cu^2+^ ions monitored at 550 nm. (e) Initial rates of Cyt *c* (10 μM) oxidation in the presence of MOF-808-His-Cu (12 μg mL^−1^) under air- and N_2_-saturated reaction conditions.

Considering the small pore-aperture of MOF-808, we hypothesized that the accessibility and hence, the reactivity of smaller proteins would be impossible. To test this, we selected Cyt *c* which is a smaller heme protein with the dimension of 2.6 nm × 3.2 nm × 3.3 nm (Fig. 3b).^106^ Cyt *c* is a mobile single electron carrier that is present in the intermembrane mitochondrial spaces. It plays a key role in shuttling electrons between complex III and cytochrome *c* oxidase (COX) in complex IV. This process of electron transfer in the electron transport chain is accompanied by the continuous pumping of protons across the membrane, which is vital for mitochondrial ATP production (Fig. 3a).^107^ In a study by Chen *et al.*^108^ they demonstrated how Cyt *c* protein enters the MOF cavities with pore sizes less than the dimensions of Cyt *c*. Recent studies by Triphathi *et al.* have shown the electrical unfolding of Cyt *c* through silicon nitride nanopores having diameters of 1.5 to 5.5 nm.^109^ Their study found that Cyt *c* could squeeze through the nanopore of 2.5 nm diameter, while fully unfolded protein threading occurred at the mouth of the nanopore with diameters 1.5 and 2.0 nm. In our study, to test the accessibility of Cyt *c* through the restricted pore, MOF-808-His-Cu could be the best choice as it also has the active center containing copper-histidine moieties, similar to the COX enzyme containing the copper redox centers with an imidazole-based ligand system.^110^

The COX activity was studied using reduced Cyt *c*, which was generated by reducing the oxidized Cyt *c* with sodium dithionite in 50 mM PB (pH 7.4) and further purified using a Sephadex G-25 desalting column. The activity is monitored using UV-vis spectroscopy, following a decrease in the Soret band at 409 nm and α-band at 550 nm, which are the characteristic peaks assigned for the oxidation of reduced Cyt *c*.^111^ In our study, reduced Cyt *c* (10 μM) in the presence of MOF-808-His-Cu (12 μg mL^−1^) showed an effective decrease in the α-band (550 nm) intensity and a blue shift of Soret band (at 409 nm) under physiologically relevant conditions (50 mM PB, pH 7.4). The decrease in intensity at 550 nm was consistent with the oxidation of Cyt *c* (Fig. 3c and S28a†), which resulted in a color change from pink to yellowish-orange (Fig. S28b†). However, minimal auto-oxidation of Cyt *c* was evident in the absence of MOF-808-His-Cu even after 60 min (Fig. S28c†). Also, MOF-808 (without Cu active sites) did not possess any COX activity under identical reaction conditions (Fig. S28d†). To validate that the COX activity displayed by MOF-808-His-Cu was not due to the leaching of Cu^2+^ ions, the catalyst was incubated in PB, followed by centrifugation. The supernatant did not display any COX activity, as evident from Fig. S29a.† In comparison to MOF-808-His-Cu, the equivalent amounts of free Cu^2+^ ions exhibited remarkably higher COX activity (Fig. 3d). Thus, it was confirmed that the COX activity was not due to the leaching of Cu^2+^ ions. Notably, when the COX activity was carried out in the N_2_-saturated buffer, an appreciable decrease in the activity was noted, revealing the role of O_2_ in the reaction (Fig. 3e).

**Fig. 4:**
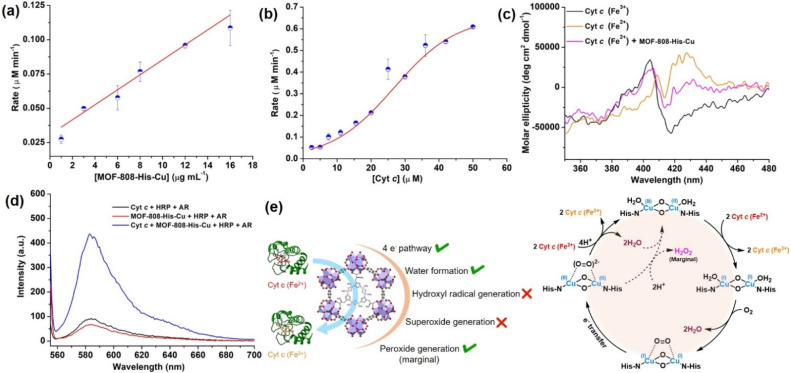
(a) Effect of varying concentration of MOF-808-His-Cu on Cyt *c* (10 μM) oxidation at pH 7.4. (b) The sigmoidal curve was obtained for the varying concentrations of Cyt *c* (2.5–50 μM) in the presence of a fixed amount of MOF-808-His-Cu (12 μg mL^−1^). (c) The oxidation of Cyt *c* (Fe^2+^) to Cyt *c* (Fe^3+^) in the presence of MOF-808-His-Cu was studied by circular dichroism (CD). (d) The AR/HRP assay for the detection of H_2_O_2_ formation during Cyt *c* oxidation in the presence of MOF-808-His-Cu. (e) The possible side reactions, ROS produced during Cyt *c* oxidation and the plausible catalytic cycle for the Cyt *c* oxidation catalyzed by MOF-808-His-Cu.

Interestingly, with the increasing concentration of nanozyme (1–17 μg mL^−1^), while keeping the Cyt *c* concentration fixed as 10 μM, we observed that the rate improved with an increase in the concentration of the catalyst, but was not equally proportional (Fig. 4a, S30a and b†). In another case, when we increased the concentration of Cyt *c* (2.5–50 μM) in the presence of 12 μg mL^−1^ of catalyst (Fig. 4b and S30c†), we observed a non-typical sigmoidal profile of the curve, distinguishing from the Michaelis–Menten kinetics observed for the natural COX.^112,113^ Initially, the activity is very low for ∼2.5–15 μM, while the catalytic efficiency gradually increases after 20 μM and proceeds further toward saturation with an increase in the concentration of Cyt *c* to about 50 μM (Fig. 4b). This is in contrast to our study on utilizing small molecule DTBC as a substrate which follows first-order kinetics with increasing catalyst concentration and a Michaelis–Menten curve upon increasing DTBC concentration (Fig. 2e). Therefore, this suggests that the oxidation of Cyt *c* follows a distinct mechanism in its interaction with the active site located within the pore. The interaction of Cyt *c* with the catalyst was confirmed by circular dichroism (CD) studies. In general, in the wavelength range of 200–300 nm, the α-helical composition of the protein is probed, while the wavelength range of 300–500 nm demonstrates the changes near the heme environment.^114^ There is a significant difference in the absorption features of reduced and oxidized Cyt *c* in the 300–500 nm region (Fig. 4c). During Cyt *c* oxidation in the presence of MOF-808-His-Cu (after 15 min incubation), no changes in the feature of the CD spectrum of reduced Cyt *c* were observed in comparison to the reduced Cyt *c* alone (control) in the 200–300 nm region (Fig. S31a and b†). Nevertheless, a significant change was observed in the region 300–500 nm, which directly reflects the changes in the heme environment and is consistent with the oxidation of reduced Cyt *c* (Fig. S31c and d†). Collectively, these results suggest that during the catalytic oxidation, the structure of Cyt *c* is not altered by MOF-808-His-Cu. This is also evident from the nature of CD spectra of oxidized Cyt *c* in the presence of MOF-808-His-Cu (Fig. S31a and c†).

To further elucidate the impact of catalyst size on the approach of the Cyt *c* protein, we evaluated the COX activity for ∼500 nm scale MOF-808-His-Cu (Fig. S32a†). Our findings, as shown in Fig. S32b and c,† indicate that the activity and rate were comparable to that observed in the nanoscale MOF-808-His-Cu. These observations suggest that irrespective of MOF-808 size, the approach of Cyt *c* remains consistent because of the identical pore size and aperture.

In mitochondria, the oxidized Cyt *c* molecules receive electrons from complex III of the electron transport chain, which further transfers electrons to COX. This follows the reduction of O_2_ to H_2_O *via* a four-electron pathway at the active site of COX.^107^ However, mitochondria also contribute to cellular oxidative stress by producing ROS such as 
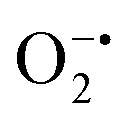
 with complex I and III being the major sites (Fig. 3a).^115^ SOD then converts the generated 
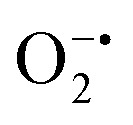
 to peroxide, which is further converted to water and O_2_ by catalase. Therefore, the antioxidant system has a tight control over ROS detoxification.^116^ Thus, it is vital to assess the generation of ROS such as 
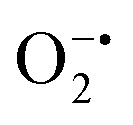
 or hydroxyl radical, during Cyt *c* oxidation. To understand the plausible mechanism for Cyt *c* oxidation in the presence of MOF-808-His-Cu, we first investigated if the addition of SOD in the reaction assay has any effect on the rate of Cyt *c* oxidation (Fig. S33a†). Under this condition, we did not observe any noticeable change in the activity, and hence, we infer that the 
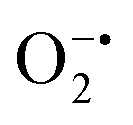
 are not generated during the reaction. Furthermore, the SOD-like activity of MOF-808-His-Cu, as discussed earlier, is complementary to Cyt *c* oxidation. Next, we evaluated the formation of H_2_O_2_ during the Cyt *c* oxidation by the AR/HRP assay (Fig. S33b†).^117^ Through the AR/HRP assay, we noticed that there was marginal peroxide formation (0.58 μM) as a result of partial reduction of O_2_, following a two-electron pathway (Fig. 4d and S33c†). This was also supported by the experiment carried out using exogenous addition of catalase during the reaction that resulted in no change in the MOF-808-His-Cu catalyzed Cyt *c* oxidation activity (Fig. S33a†). Finally, we tested if hydroxyl radicals are generated from the marginally produced H_2_O_2_. The formation of hydroxyl radicals was probed by TPA. No apparent emission was observed in the catalytic system for MOF-808-His-Cu + Cyt *c* + TPA. When Cyt *c* oxidation was performed with an equivalent amount of Cu^2+^ in the presence of TPA, an intense fluorescence emission at 425 nm was observed (Fig. S33d†). Thus, from this experiment, it can be concluded that no hydroxyl radical species are generated during the oxidation of Cyt *c* in the presence of MOF-808-His-Cu. Through a series of experiments and characterization of MOF-808-His-Cu, we have elucidated a plausible catalytic mechanism (Fig. 4e). Overall, in a major pathway, the oxidation of Cyt *c* by MOF-808-His-Cu results in the reduction of O_2_ to water by the transfer of four-electrons from four Cyt *c* molecules. The minor pathway, *i.e.*, production of H_2_O_2_ results from the two-electron reduction of O_2_ molecule as shown in Fig. 4e. However, this is in contrast to the catechol oxidase-like activity of MOF-808-His-Cu using DTBC as a substrate that results in the generation of H_2_O_2_. This could be due to the difference in electron transfer mechanisms that depend on the type of substrates involved in the activity.

This work mainly focuses on studies pertaining to the selectivity of nanozymes and evaluating critical factors that can compromise applications. Therefore, the interaction of small proteins with porous structure-based nanozymes requires careful attention. The detailed investigation of the mechanism by which Cyt *c* accesses the active site of MOF-808-His-Cu will be discussed in the follow-up work.

## Conclusions

The growing interest in nanozymes highlights their potential in various applications. However, limitations associated with their specificity and selectivity require careful attention. Despite considerable efforts to develop artificial enzymes to achieve specificity comparable to natural enzymes, it remains a long-standing challenge, necessitating further advancement in this area. MOFs have gained significant attention as artificial enzyme mimics since they offer tuning of the structural features within a single construct. The 3D structure with nanoconfinement and exactly uniform repeating pores, along with the substrate channels, can play an influential role in enhancing the catalytic performance. Furthermore, modulating the catalytic microenvironment with ligands similar to natural enzyme counterparts, and substrate recognition sites can help improve the structure–activity relationship of the nanozyme. The multi-enzymatic behavior can, in turn, result in a decrease in catalytic efficiency, thus achieving substrate selectivity, and single-enzyme mimic features can play a vital role in improving the catalytic efficiency with high specificity. Furthermore, as nanozymes offer promising therapeutic and biotechnological prospects, the antagonistic multienzyme mimetic activities and lack of substrate selectivity can negatively affect their intended applications. Through our investigation, we have shown the significance of pore-engineering strategies of MOFs in achieving selectivity in enzyme mimics. The biomimetic active site (bis-(μ-oxo) dicopper) within the pore of MOF-808 exhibited an impressive substrate-selective oxidase activity by predominantly oxidizing the catechol-like substrates. This substrate selective oxidase mimic, wherein the approach and the reactivity of the substrates are tightly controlled by the small pore-opening (1.4 nm) as inspired by the opening of enzyme binding pockets, makes it distinct from the other oxidase mimic in achieving substrate selectivity. Despite this well-regulated access to the active sites and effective prevention of approach of DNA to the active site and its ROS-mediated damage, the small proteins such as Cyt *c* having dimensions larger than the pore-opening of MOF-808 can access the active site and compromise the selectivity of the nanozyme. As it is important to acknowledge this limitation of MOF-808-His-Cu, our studies on the detailed mechanism of interaction and the reactivity of this flexible protein (Cyt *c*) will be discussed in the follow-up work. The unusual approach of redox protein towards pore-engineered nanozymes could hamper several redox and energy metabolism pathways and influence unintended reactions, thus, it warrants special attention. This study significantly points to setting guidelines for the development of next-generation enzyme mimetics with improved strategies, thereby advancing their potential use in several biological applications.

## Data availability

The data supporting this article have been included as part of the ESI.†

## Author contributions

RVM and AAV conceived the idea. RVM synthesized, functionalized and characterized the MOFs, and performed experimental work. APF assisted in kinetic studies and helped in establishing the mechanism. AAV supervised the project. All the authors wrote and edited the manuscript.

## Conflicts of interest

There are no conflicts to declare.

## Supplementary Material

SC-015-D4SC02136C-s001
