# Supplementary material for: Approach of a small protein to the biomimetic bis-(μ-oxo) dicopper active-site installed in MOF-808 pores with restricted access perturbs substrate selectivity of oxidase nanozyme
Source: Chem Sci. 2024 Jun 10;15(28):10810–22. doi: 10.1039/d4sc02136c (PMC11253172; doi:10.1039/d4sc02136c)
Supplement: SC-015-D4SC02136C-s001 [file SC-015-D4SC02136C-s001.pdf]

Electronic Supplementary Material (ESI) for Chemical Science.
This journal is © The Royal Society of Chemistry 2024

Approach of a small protein to the biomimetic bis-(μ-oxo) dicopper active-site installed in MOF-808 pores with restricted access perturbs substrate selectivity of oxidase nanozyme

Rasmi V. Morajkar, [ab] Adarsh P. Fatrekar [ab] and Amit A. Vernekar *[ab]

[a] *Inorganic and Physical Chemistry Laboratory, CSIR-Central Leather Research Institute, Chennai 600020, Tamil Nadu, India.*

[b] *Academy of Scientific and Innovative Research (AcSIR), Ghaziabad-201002, India.*

Table of Contents

Materials and Methods ……………………………………………………………………**S2-S11**
Figure S1. The bis-(μ-oxo) dicopper active site in the pore of MOF-808……………….**S12**
Figure S2. SEM of MOF-808, MOF-808-His and HRTEM images of MOF-808-His-Cu..**S12**
Figure S3. N_2 adsorption-desorption isotherm of MOF-808-His-Cu……………………. **S13**
Figure S4. UV-vis DRS spectra of MOF-808-His-Cu……………………………………. **S13**
Figure S5: ^{1}H NMR spectrum of MOF-808………………………………………………. **S14**
Figure S6: ^{1}H NMR spectrum of MOF-808-His……………………………………………. **S15**
Figure S7: ^{1}H NMR spectrum of MOF-808-His-Cu…………………………………….... **S16**
Figure S8: XPS spectra of MOF-808-His-Cu…………………………………………….. **S16**
Figure S9: Cu K-edge XANES and, EXAFS spectra with fitting results……………….. **S17**
Figure S10: Kinetic studies for the DTBC oxidation……………………………………… **S18**
Figure S11: Evaluating stability MOF-808-His-Cu and the catalytic cycle for DTBC oxidation…………………….. ………………………………………………………….. **S19**
Figure S12: Quercetin oxidation in presence of MOF-808-His-Cu…………………….. **S20**
Figure S13: (+)-Catechin oxidation in presence of MOF-808-His-Cu………………….. **S20**
Figure S14: L-ascorbic acid oxidation in presence of MOF-808-His-Cu……………….. **S21**
Figure S15: Evaluation of D-glucose oxidation…………………………………………... **S22**
Figure S16: Evaluation of Xanthine oxidation…………………………………………….. **S23**
Figure S17: Evaluation of Uric acid oxidation…………………………………………….. **S24**
Figure S18: Evaluation of NADH oxidation……………………………………………….. **S24**
Figure S19: Evaluation of NADPH oxidation……………………………………………... **S25**
Figure S20: Evaluation of L-Tyrosine oxidation…………………………………………... **S25**
Figure S21: Evaluation of substrate selectivity of MOF-808-His-Cu…………………… **S26**
Figure S22: Evaluation of oxidase activity using AR at pH 7.4………………………... **S27**
Figure S23: Evaluation of peroxidase activity using AR at pH 7.4…………………….. **S28**
Figure S24: Evaluation of catalase activity at pH 7.4…………………………………… **S29**
Figure S25: Evaluation of glutathione peroxidase activity at pH 7.4………………….. **S29**
Figure S26: Evaluation of superoxide dismutase activity at pH 7.4…………………… **S30**
Figure S27: Fenton reaction and DNA damage studies ………………………………… **S31**
Figure S28: Cyt *c* oxidase (COX) activity of MOF-808-His-Cu………………………… **S32**
Figure S29: Evaluating leaching of Cu^{2+} ions …………………………………………… **S33**
Figure S30: Kinetic studies of COX activity of MOF-808-His-Cu………………………. **S33**
Figure S31: CD spectra for COX activity of MOF-808-His-Cu…………………………... **S34**
Figure S32: Effect of size of MOF-808-His-Cu on COX activity………………………... **S35**
Figure S33: Experimental studies for the mechanism for COX activity……………….. **S36**

Table S1: EXAFS fitting results of MOF-808-His-Cu……………………………………..S17
Table S2: Catalytic parameters for reported oxidase nanozymes and MOF-808-His-Cu towards catechol substrate. ………………………………………………………..………...….. S18

References: …………………………………………………………………………………..S37

Materials and Methods

Chemicals

Zirconium tetrachloride ($ZrCl_4$) and 3,5- di-tert-butyl catechol (DTBC) (>98%), were purchased from Tokyo Chemical Industry (India) Pvt. Ltd. Acetonitrile (ACN), >99.5% purity, deuterium oxide (D_2O), 99%, dimethyl sulfoxide-d_6 (DMSO-d_6), 99.8%, deuterium chloride solution (DCl), 35wt% in D_2O, >99 atom % D, (+) - Catechin, 98%, (Hydrate minimum), Quercetin dihydrate, D-(+)-Glucose, Glutathione reductase (GRx) from baker's yeast (*S. cerevisiae*), Deoxyribonucleic acid sodium salt from calf thymus (DNA), Xanthine (XA), 99%, Superoxide dismutase (SOD) from bovine liver, Xanthine oxidase (XO) from bovine milk, catalase from bovine liver, Cell proliferation reagent WST-1, terephthalic acid (TPA), Ampliflu red (10-Acetyl-3,7-dihydroxyphenoxazine) (AR), >98% were purchased from Sigma-Aldrich Chemicals Pvt. Ltd.

N, N-dimethylformamide (DMF), 99%, L-Histidine, 99%, Cuprous Iodide, 99% (CuI), 3,3,5,5-tetramethylbenzidine dihydrochloride anhydrous, 99% (TMB), hydrogen peroxide (H_2O_2) solution 30%, L-ascorbic acid (AA), 99.7 %, L-Tyrosine (Ty),99%, sodium phosphate dibasic anhydrous, 99%, sodium phosphate monobasic monohydrate, 99%, tris hydrochloride (Tris HCl), 98% Glucose oxidase (GO) ex. *Aspergillus niger*, β-Nicotinamide adenine dinucleotide phosphate tetrasodium salt (reduced) (β-NADPH.Na_4) extra pure 98%, β-Nicotinamide adenine dinucleotide (reduced) disodium salt (NADH), Glutathione reduced (GSH), 99%, Cytochrome *c* (Cyt *c*) (oxidized) (Type1) ex. Horse Heart, extra pure, sodium dithionite ($Na_2S_2O_4$) extra pure 85%, Peroxidase ex. Horseradish (type 3) (HRP) were purchased from SRL Chemicals Pvt. Ltd. Sephadex G-25 coarse was purchased from Pharmacia Fine Chemicals AB, Sweden. Uric acid (UA), 99%, was purchased from Alfa Aesar Pvt. Ltd.

Characterization methods

Scanning electron microscopy (SEM) imaging was performed using a Field emission scanning electron microscope, TESCAN CLARA. Transmission electron microscopy (TEM) images were recorded on the JEOL JEM 2100 Plus electron microscope by drop-

casting finely dispersed samples on a copper grid. Powder X-ray diffraction (XRD) analysis was performed on the Rigaku mini flex-II desktop (Cu Kα 1.5406 Å radiation). X-ray photoelectron spectroscopy (XPS) was recorded on a Thermo Scientific, MultiLab 2000 with monocromated Al Kα radiation (1486.6 eV) operated at 15 KV. Ultraviolet-visible-diffuse reflectance (UV-DRS) spectra were recorded on Cary series UV-Vis-NIR Spectrophotometer, Agilent. Proton Nuclear magnetic resonance (^{1}H NMR) spectra were recorded on a 400 MHz Nuclear magnetic resonance spectrometer, Bruker Avance III HD. Gas adsorption analysis was performed on a 3Flex adsorption analyzer Version 3.0. Cu K-edge EXAFS measurements were carried out using a RIGAKU R-XAS laboratory spectrometer using transmission mode. To process and fit the data, the Athena and Artemis modules of the Demeter software package were employed. Circular dichroism (CD) spectra were recorded on a Jasco J-815 CD spectrometer.

Synthesis of MOF-808

Nanoscale MOF-808 was synthesized following the reported protocol with slight modification.[1,2] In a round bottom flask, $ZrCl_4$ (725 mg) was mixed with DMF (23 mL) and formic acid (23 mL) and sonicated for 30 min. Then, 1,3,5-benzene tricarboxylic acid (500 mg) was added to the mixture and further sonicated for 10 min. Thereafter, the solution was refluxed for 3 h under stirring at 120 °C. The white powder was collected by centrifugation (8000 rpm, 10 min) and washed with DMF (3 x 50 mL) over 24 h, followed by acetone wash (3 x 50 mL) over 24 h. The as-synthesized nanoscale MOF-808 was vacuum-dried at room temperature (25 °C).

Functionalization of MOF-808 with L-Histidine

In a 30 mL vial L-histidine (600 mg) was dissolved in water (7 mL) and heated to 85 °C in an isothermal oven. MOF-808 (130 mg) was suspended and sonicated, followed by the addition of a saturated L-histidine solution. Later, the reaction mixture was heated overnight in an isothermal oven at 85 °C. The supernatant was carefully removed while the reaction mixture was hot, to avoid recrystallization of L-histidine. White powder was collected by centrifugation (8,000 rpm, 5 min), and washed with water (10 mL × 5) over 3 days, followed by acetone washings for three days (10 mL × 5). The as-synthesized MOF-808-His was dried under a dynamic vacuum at room temperature (25 °C).

Post-ligand metalation of MOF-808-His with copper

To a suspension of MOF-808-His (74 mg) in ACN (2 mL) in a 30 mL glass vial, a solution of CuI (46.7 mg) in ACN (7 mL) was added dropwise under constant stirring (500 rpm). The mixture was allowed to stir for three days at 25 ^{o}C. The solid green powder obtained after the reaction was collected by centrifugation (8,000 rpm, 5 min) and washed five times with ACN (15 mL× 5) over three days. The compound MOF-808-His-Cu was dried under a vacuum at 25 ^{o}C.

^{1}H NMR spectrum

The solutions for ^{1}H NMR spectra were prepared by digesting the synthesized MOF samples using a mixture of deuterated solvents. Around 5 mg of the samples were digested and dissolved using a mixture of DMSO-d_6 (600 μL), deuterium chloride (50 μL) and, D_2O (20 μL) as reported earlier.[2,3] The spectral measurements for all the samples were recorded under the same experimental conditions. The peaks for minor impurities are attributed to residual solvents in the MOF.

XANES and EXAFS

Cu K-edge absorption spectroscopy measurement was carried out using a RIGAKU R-XAS laboratory spectrometer, equipped with a 3 kW X-ray source and Ge (220) monochromator. Data was recorded in the transmission mode using a gas-filled ionization chamber before the sample and a scintillation detector after the sample. The edge energy was calibrated using commercially obtained Cu & CuO powders (Sigma-Aldrich, 99.99% purity) as the standards. The absorbers prepared using sample powders were adjusted for their thickness to obtain an optimal absorption jump. Experiments were performed at room temperature. The pre-edge background subtraction and normalization of the recorded spectra were performed using the Athena module of the Demeter software package. The energy corresponding to the absorption edge (E_0) was determined from the first derivative of the normalized spectra in the near-edge (XANES) region. EXAFS spectra were recorded up to 800 eV above the edge and yielded good data in the k-space up to ~ 11 Å^{-1}. EXAFS fitting was carried out using the Artemis module of the Demeter software package.

Determination of catechol oxidase activity

To examine the catechol oxidase activity, DTBC, quercetin, and (+)-catechin were selected as the substrates. The reaction was carried out with MOF-808-His-Cu (12 μg mL^{-1}) in 50 mM phosphate buffer (PB), pH 7.4 at 25 ^{o}C and to increase the solubility of the substrate ACN (200 μL) was added. The final concentrations of DTBC, quercetin, and (+) catechin were 200, 30, and 200 μM, respectively. The oxidation of AA (125 μM) in the presence of MOF-808-His-Cu (12 μg mL^{-1}) at pH 7.4, 25 ^{o}C was monitored using UV-vis spectroscopy. It showed a decrease in absorbance at 265 nm with time. To further confirm the formation of dehydroascorbic acid as the oxidation product of AA, it was probed using OPD.[4] 125 μM of AA was incubated with MOF-808-His-Cu in PB for 10 min at 25 ^{o}C. Later, the resulting mixture was treated with 2 mM OPD and further incubated for another 15 min. The fluorescence spectrum was recorded at an excitation wavelength of 350 nm.

Stability of MOF-808-His-Cu under different conditions

To determine that the oxidation of DTBC was not due to the free Cu^{2+} ions leached from MOF-808-His-Cu, 1 mg mL^{-1} of MOF-808-His-Cu was incubated in 50 mM PB (pH 7.4) for 60 min. Upon incubation, it was centrifuged and the supernatant was checked for the DTBC oxidation at pH 7.4. The reaction was monitored at 415 nm using UV-vis spectroscopy in time-driven mode.

Further, the stability of MOF-808-His-Cu under different pH and temperature conditions was evaluated. The pH stability was checked using a known amount of MOF-808-His-Cu was incubated in 50 mM sodium acetate buffer (for pH 4-5) and 50 mM PB (for pH 6-8) for 60 min at 25 ^{o}C and centrifuged to recover the catalyst. Later the catalyst was re-dispersed in distilled water and 12 μg mL^{-1} was used to check the oxidase activity. The oxidation of DTBC in the presence of MOF-808-His-Cu was performed at pH 7.4 in kinetics mode using a UV-Vis spectrophotometer. To check the stability of MOF-808-His-Cu under different temperature conditions, the catalyst dispersed in distilled water was incubated at different temperatures of 25 ^{o}C, 60 ^{o}C, and 80 ^{o}C for 15 min. The oxidase activities of incubated MOF-808-His-Cu at various temperatures were checked at pH 7.4 in kinetics mode using a UV-Vis spectrophotometer.

Evaluation of H_2O_2 formation during DTBC oxidation by MOF-808-His-Cu

The production of H_2O_2 during DTBC oxidation catalyzed by MOF-808-His-Cu was evaluated using TMB/ HRP assay. The oxidation of DTBC (200 μM) catalyzed by MOF-808-His-Cu (12 μg mL^{-1}) was performed in 50 mM PB (pH 7.4) at 25 ^{o}C. Upon incubation for 30 min, the reaction mixture was centrifuged and the supernatant was added in a vial containing TMB (0.4 mM), and HRP (1 U) in 50 mM sodium acetate buffer (pH 4). The control reaction was performed in the absence of MOF-808-His-Cu under similar reaction conditions. The oxidation of TMB was monitored at 650 nm using UV-vis spectroscopy.

Evaluation of substrate selectivity of MOF-808-His-Cu

Glucose oxidase assay: In a typical glucose oxidase assay, the catalytic oxidation of glucose generates H_2O_2 which is probed by HRP to oxidize chromogenic redox species such as TMB.[5] A 1mL reaction composed of 1M glucose containing 12 μg mL^{-1} MOF-808-His-Cu in a 50 mM PB (7.4) was incubated for 5 min at room temperature (25 ^{o}C). Later, 20 μL HRP (0.1 μg mL^{-1}) and 0.4 mM TMB were added and UV-vis spectra were recorded after 30 min incubation. As a positive control, the reaction was carried out under the same condition, in the presence of 1 U mL^{-1} of GO.

Glucose consumption test: Oxidation of glucose by MOF-808-His-Cu was assessed based on the estimation of glucose using a 3,5-dinitrosalicylic acid (DNS) assay. All the solutions for the DNS assay were prepared as mentioned in the literature.[6] The DNS reagent was prepared using 500 mg of DNS dissolved in 25 mL of distilled water and heated at 80 ^{o}C (5 min) for complete dissolution. The solution was allowed to cool to room temperature, and 10 mL of potassium sodium tartrate tetrahydrate solution (3 g dissolved in 10 mL of 2 N NaOH) was added. This solution was further diluted to a total volume of 50 mL using distilled water.

According to the assay, 1 mL of DNS reagent and 1 mL of the sample (distilled water in case of blank) were taken in a test tube, and to each test tube 3 mL of distilled water was added. The solution was heated in a water bath (at 90 ^{o}C) for 15 min and immediately cooled in ice-cold water to stop the reaction. From this, 1 mL of the reaction mixture was taken in a cuvette, and its absorbance was measured at 540 nm. The standard calibration curve for the DNS assay was plotted using different concentrations of glucose (0-1000 μM). To evaluate the oxidation of glucose by MOF-808-His-Cu, 3 mM glucose as control and 3 mM glucose in the presence of 72 μg mL^{-1} MOF-808-His-Cu was performed at pH 7.4, 25 ^{o}C for 60 min. The reaction mixture was centrifuged and, 1 mL of the supernatant was taken in a

test tube and DNS assay was performed as mentioned above. It should be noted that the final concentration of glucose for estimation using the DNS method should be close to 600 μM upon final dilution to 5 mL. For quantification of glucose in reaction (catalyzed by MOF-808-His-Cu) the absorbance was compared with the control reaction containing the same concentration of glucose and was further verified using the calibration curve.

Xanthine oxidase assay: The typical oxidation of XA results in UA and H_2O_2 formation. The H_2O_2 produced can be probed by the AR/ HRP assay.[7] The reaction mixture containing 500 μM of XA, AR (5 μM), and HRP (2.5 U mL^{-1}) in PB (50 mM, 7.4) was reacted with MOF-808-His-Cu (12 μg mL^{-1}) and incubated for 60 min at 25 ^{o}C. Fluorescence spectra were recorded using an excitation wavelength of 550 nm and an excitation slit width of 2.5 nm and an emission slit width of 5 nm. As a positive control XO (16.8 mU mL^{-1}) enzyme was used under the same reaction condition which resulted in an intense pink color with emission at 583 nm within 5 min.

Xanthine consumption test: XA (100 μM) oxidation in the presence of 12 μg mL^{-1} of MOF-808-His-Cu in 50 mM PB (7.4) was performed at 25 ^{o}C for 60 min. The reaction was monitored (λ_{max} 270 nm) by UV-vis spectroscopy, which revealed no change in the absorbance of XA at 270 nm. A control reaction was performed without MOF-808-His-Cu.

Uric acid assay: The oxidation of UA (50 μM) with 12 μg mL^{-1} of MOF-808-His-Cu in 50 mM PB (7.4) was performed at 25 ^{o}C.[8] The reaction monitored (λ_{max} 290 nm) by UV-vis spectroscopy over time showed no change in the absorbance of UA.

NADH and NADPH: The oxidation of both 0.2 mM NADH and NADPH with 12 μg mL^{-1} MOF-808-His-Cu in 50mM PB (7.4) at 25 ^{o}C was monitored at wavelength 340 nm by UV-vis spectroscopy.

L-Tyrosine: 500 μM of Ty was incubated for 60 min with MOF-808-His-Cu (12 μg mL^{-1}) in a 50 mM PB buffer (7.4) at room temperature (25 ^{o}C). To confirm the dityrosine formation, fluorescence spectra were recorded using an excitation wavelength of 300 nm and keeping a slit width of 20 nm. There was no emission observed at about 410 nm which is characteristic of the dityrosine formation.

Evaluation of substrate selectivity of MOF-808-His-Cu in the presence of multiple substrates

The UV-vis spectra of 1 mL reaction mixture containing 100 μM DTBC, glucose, XA, Ty, and, UA in the presence of 12 μg mL^{-1} MOF-808-His-Cu was monitored for 28 min. Additionally, the absorbance at 415 nm was monitored in a time-drive mode for the reaction mixture containing 100 μM of DTBC, glucose, XA, Ty and, UA in presence of 12 μg mL^{-1} MOF-808-His-Cu. A control reaction without MOF-808-His-Cu was also carried out under similar reaction conditions.

Determination of selectivity of enzyme-like activity

Oxidase and Peroxidase-like activity: The oxidase and peroxidase-like activity of MOF-808-His-Cu at pH 7.4 was evaluated under physiological pH conditions using AR as a substrate (suitable to use at neutral pH). The oxidase-like activity was performed using 1 μM of AR in 50 mM PB (pH 7.4) containing 12 μg mL^{-1} of MOF-808-His-Cu at 25 ^{o}C. The reaction was incubated for 30 min and fluorescence spectra (slit width = 5 nm) of the samples were recorded (λ_{ex} = 550 nm/ λ_{em} = 583 nm). Similarly, the peroxidase-like activity was monitored using 1 μM of AR with MOF-808-His-Cu (12 μg mL^{-1}) in the presence of 1 mM H_2O_2 at pH 7.4. As a positive control, the peroxidase-like activity was performed using 1 μM AR, 1 mM H_2O_2 in the presence of HRP (2 U) at pH 7.4. The reaction was incubated for 30 min and fluorescence spectra (slit width = 5 nm) of the samples were recorded (λ_{ex} = 550 nm/ λ_{em} = 583 nm).

Catalase-like activity: The catalase-like activity of MOF-808-His-Cu was measured by monitoring the decomposition of H_2O_2 to water at 240 nm (molar extinction coefficient of H_2O_2 at 240 nm = 39.4 M^{-1} cm^{-1}) using a UV-Vis spectrophotometer. The reactions were carried out at pH 7.4 (12 μg mL^{-1}) at 25 ^{o}C in presence of MOF-808-His-Cu (12 μg mL^{-1}) at fixed H_2O_2 (5 mM) concentration under time-drive mode.

Glutathione peroxidase (GPx)-like catalytic activity: The GPx-like activity of MOF-808-His-Cu was performed using the classical glutathione reductase (GR)-coupled assay on a UV-vis spectrophotometer. The reaction was monitored by following the decrease in the concentration of NADPH at 340 nm in kinetics mode.[9] For the assay, the reactants were added in the following sequence, MOF-808-His-Cu (12 μg mL^{-1}), GSH (2 mM), NADPH (0.4 mM), GR (1.7 U mL^{-1}), H_2O_2 (240 μM) in 1 mL of 50 mM PB (pH 7.4), and reaction rate was followed for 300 seconds at 25 ^{o}C.

Superoxide dismutase (SOD) activity: The SOD like-activity was evaluated by catalyzing the conversion of $O_2^{-\bullet}$ to H_2O_2 and O_2. The $O_2^{-\bullet}$ is generated by the reaction between XA

and XO. The $O_2^{-\bullet}$ further reduces the WST-1 (2-(4-iodophenyl)-3-(4-nitrophenyl)-5-(2,4-disulfophenyl)-2H-tetrazolium, monosodium salt) to formazan (spectrophotometrically detectable at 440 nm). The amount of $O_2^{-\bullet}$ generated during the reaction is proportional to the amount of formazan produced.[10] For the SOD-like-activity of MOF-808-His-Cu, 500 μM of XA was reacted with XO (33.6 mU mL^{-1}) in presence of 12 μg mL^{-1} MOF-808-His-Cu at 25 ^{o}C. The control reactions in the absence of MOF-808-His-Cu were also performed.

Fenton-like reaction: The Fenton-like reaction was performed using 0.5 mM TPA, MOF-808-His-Cu (50 μgmL^{-1}), $CuSO_4.5H_2O$ and 1 mM H_2O_2. The reaction mixture was incubated for 30 min at 25 ^{o}C. The fluorescence spectra were recorded with an excitation wavelength of 315 nm with a slit width of 5 nm.

DNA cleavage: In a typical assay of 0.5 mL in PB (50 mM, pH 7.4,) containing Calf thymus DNA (10 μg mL^{-1}), H_2O_2 (1 mM), and catalyst. The catalyst amount was fixed as MOF-808 (12 μg mL^{-1}), MOF-808-His-Cu (12 μg mL^{-1}), and, $CuSO_4.5H_2O$ (13.4 μM). The reaction mixture was incubated for 2 h at 25 ^{o}C in the dark. These samples (containing 0.5 μg mL^{-1} EtBr) were loaded and resolved on 1% agarose gel electrophoresis at 100 V for 30 min. Finally, the resulting image of the gel was captured and recorded on the Syngene Gel Doc system. Similarly, as a control experiment, the DNA cleavage studies were also performed without H_2O_2. The intensity of bands on the gel was analyzed using Image J software and the relative band intensity was graphically represented.

Probing interactions of Cyt *c* with MOF-808-His-Cu

The oxidized Cyt *c* (Fe^{3+}) was reduced to ferrous (Fe^{2+}) Cyt *c* (reduced) by adding sodium dithionate ($Na_2S_2O_4$). This reduced Cyt *c* solution mixture was passed through a Sephadex G-25 desalting column using 50 mM PB (pH 7.4) to remove the unreacted $Na_2S_2O_4$.[11]

The Cyt *c* oxidase (COX) activity of MOF-808-His-Cu was monitored by a UV-vis spectrophotometer in absorbance mode. The reaction mixture contained 10 μM of Cyt *c* in 50 mM PB (7.4) with 12 μg mL^{-1} of MOF-808-His-Cu. Also, time-based kinetics were performed by monitoring the decrease in the absorbance at 550 nm at 25 ^{o}C. The initial rate was calculated from the kinetic studies performed for 300 s. Further, the kinetic studies were performed by changing the catalyst concentration (1-16 μg mL^{-1}) and keeping a fixed concentration of Cyt *c* (10 μM). Also, the kinetic studies were performed for the change in Cyt *c* concentration (2.5 - 50 μM) by keeping a fixed concentration of MOF-808-His-Cu as 12 μg mL^{-1}.

To study the COX activity of MOF-808-His-Cu under Air- and N_2 saturated conditions:

In this study, all the solutions including the PB (50 mM , 7.4), catalyst and the reduced Cyt *c* stock solution were saturated with N_2. Using the N_2-saturated solutions of 10 μM of Cyt *c* in presence of 12 μg mL^{-1} MOF-808-His-Cu at pH 7.4 (50mM PB), the time-based absorbance was monitored at 550 nm for 300 seconds. Similarly, the reaction was performed in the air-saturated condition and the initial rates were compared to check the role of O_2 during oxidation.

CD spectroscopic studies

The CD studies were carried out in a JASCO- J-815 CD spectropolarimeter using a 0.1 cm path length quartz cuvette, scanning speed of 100 nm /min, and data pitch of 0.2 nm. Three full wavelength scans were recorded and the spectra were averaged. The CD spectra were recorded within two wavelength ranges: 190-300 nm and 300-500 nm. The baseline correction was done by using 20 mM of PB (pH 7.4) as the blank solution. CD measurements were carried out to determine the possible interaction and binding of Cyt *c* to MOF-808-His-Cu.[12] The CD spectra of 50 μM of ferrous (Fe^{2+}) and ferric (Fe^{3+}) Cyt *c* in the presence and absence of 50 μg mL^{-1} MOF-808-His-Cu were recorded. The spectra were recorded after mixing for 15 min at 25 ^oC.

The Cyt *c* oxidation by MOF-808-His-Cu in presence of SOD and catalase enzyme:

To evaluate the generation and the effect of hydroxyl radical and $O_2^{\bar{}\bullet}$ during Cyt *c* oxidation catalyzed by MOF-808-His-Cu, we performed the assay by adding catalase and SOD enzyme, respectively. To study this, the initial rate of Cyt *c* oxidation in presence of MOF-808-His-Cu, and upon the addition of catalase and SOD were compared. The kinetics at 550 nm was monitored for a reaction mixture containing 10 μM of Cyt *c* in presence of 12 μg mL^{-1} MOF-808-His-Cu at pH 7.4 (50 mM PB) at 25 ^oC. Similarly, the initial rate was calculated for the assay containing 10 μM of Cyt *c* + MOF-808-His-Cu (12 μg mL^{-1}) in presence of catalase (1 U mL^{-1}) and SOD (1 U mL^{-1}) at pH 7.4 for evaluating the influence of hydroxyl radical and $O_2^{\bar{}\bullet}$, respectively.

Detection of H_2O_2 during Cyt *c* oxidation: The formation of H_2O_2 during Cyt *c* oxidation was probed using AR/HRP assay using fluorescence spectroscopy.[7] All the measurements were carried out using an excitation wavelength of 550 nm and a slit width of 5 nm. The

assay was performed using 10 μM of Cyt *c*, AR (10 μM), and HRP (5 U mL^{-1}) in the presence of 50 μg mL^{-1} MOF-808-His-Cu at pH 7.4 (50 mM PB) at 25 ^{o}C . The reaction was incubated for 60 min and fluorescence spectra of the samples were recorded. A calibration plot with varying concentrations of H_2O_2 using the AR/ HRP assay under similar conditions as maintained in the above reaction was performed using fluorescence spectroscopy (λ_{ex} = 550 nm/ λ_{em} = 583 nm).

Detection of hydroxyl radical during Cyt *c* oxidation: The hydroxyl radicals are generally probed by terephthalic acid (TPA) which results in an intense emission at 425 nm (due to the formation of hydroxy TPA) upon excitation at 315 nm.[13] To determine the generation of hydroxyl radical generation during Cyt *c* oxidation, 0.5 mM TPA, and 10 μM Cyt *c* were incubated with 50 μg mL^{-1} of MOF-808-His-Cu for 30 min at 25 ^{o}C. Similarly, the reaction was performed using an equivalent amount of $CuSO_4.5H_2O$ instead of MOF-880-His-Cu. The fluorescence spectra were recorded with an excitation wavelength of 315 nm and a slit width of 20 nm.

Protein images and MOF-808 structures

The images of proteins, GO, GR, XO, HRP and Cyt *c*, were created by using Chimera software and PDB entries 1CF3, 1GRE, 1FIQ, 1HCH, and 1OCD, respectively. The MOF-808 structure was created using a CIF file (deposition number 1002672) deposited in the Cambridge Crystallographic Data Centre (CCDC).

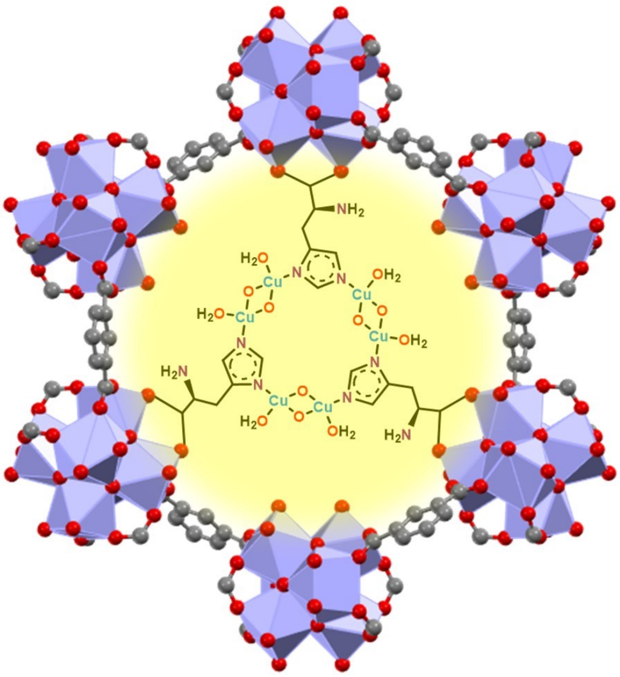

Figure S1: The adamantane pore of MOF-808 bearing the biomimetic bis-(μ-oxo) dicopper active site.

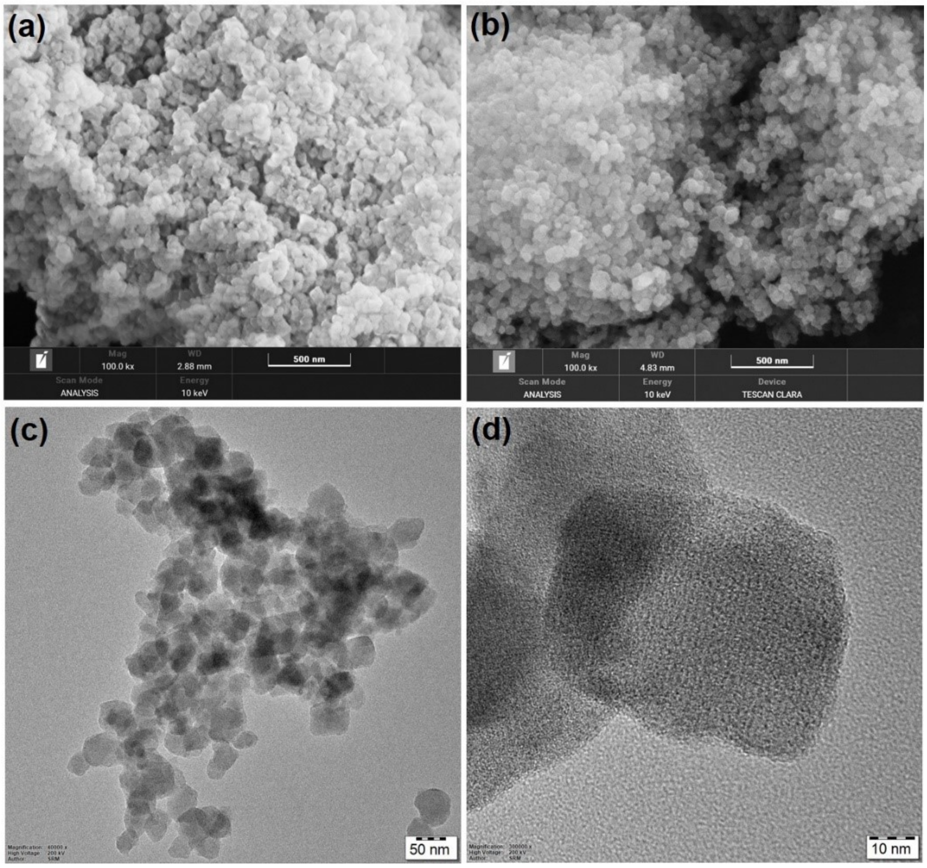

Figure S2: SEM images of **(a)** MOF-808 and **(b)** MOF-808-His. **(c-d)** HRTEM images of MOF-808-His-Cu.

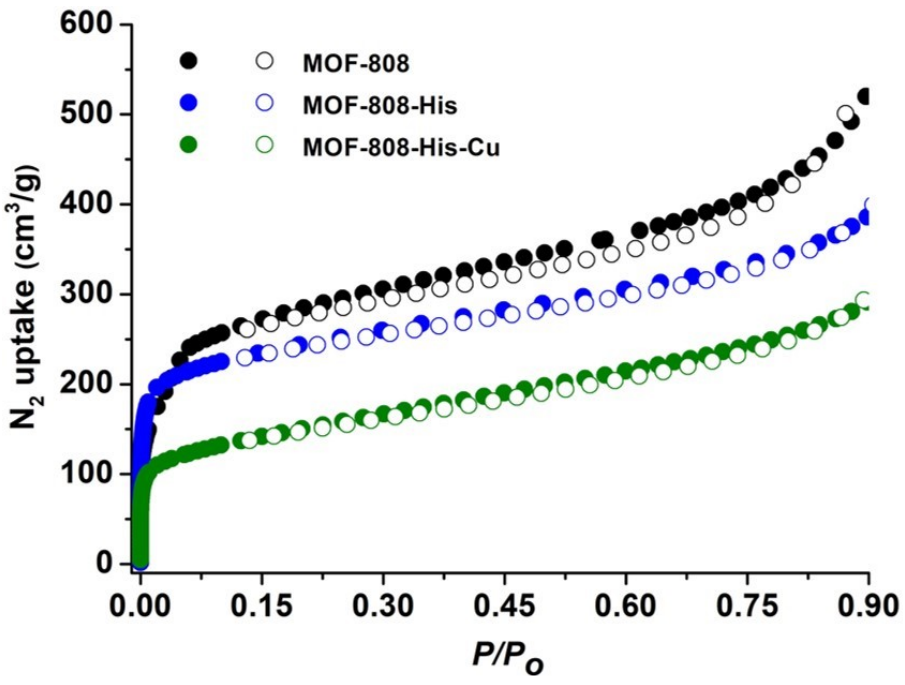

Figure S3: N_2 adsorption-desorption isotherms recorded at 77 K demonstrating a decrease in BET surface area on pore functionalization of MOF-808 with L-histidine and copper.

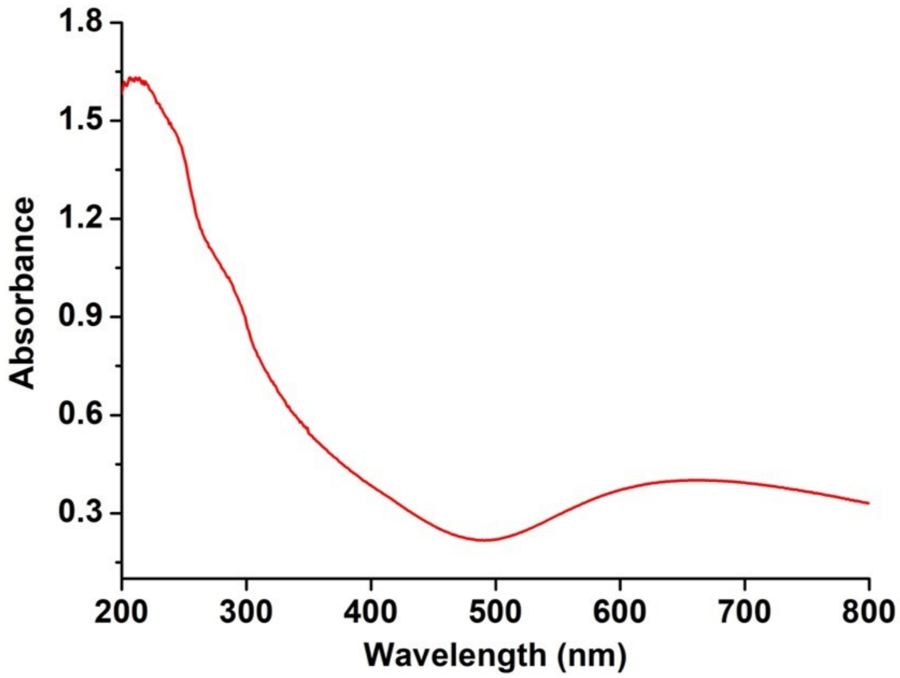

Figure S4: UV-vis DRS spectrum of MOF-808-His-Cu.The charge transfer (CT) bands at ~300 – 400 nm are due to the oxygen to copper CT in MOF-808-His-Cu and the broad band in the region of 500 – 800 nm arises due to d-d transition.[14] The band at ~290 nm accounts for the n−π* transition of imidazole moieties. The evident absorption band centered at ~650 nm marks the Cu-(μ-oxo)-Cu species in MOF-808-His-Cu.[15]

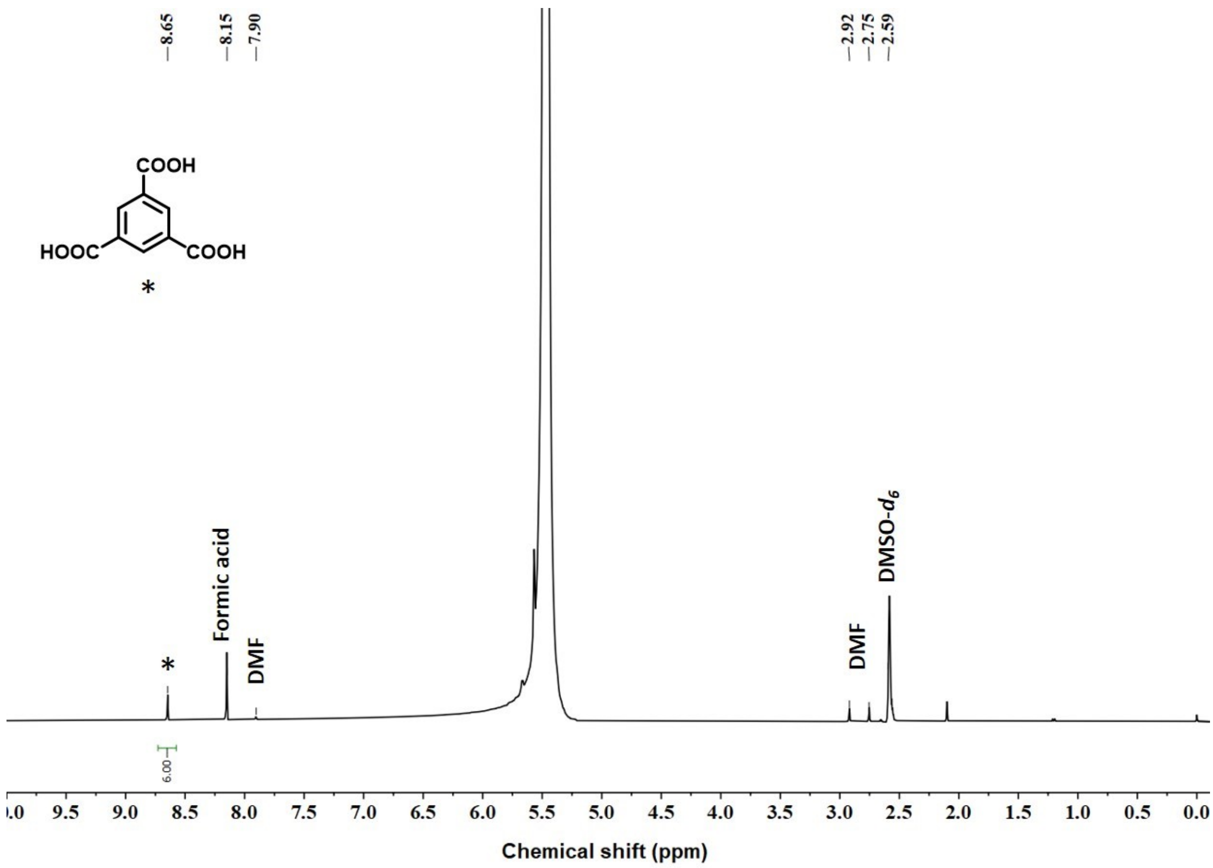

Figure S5: ^{1}H NMR spectrum of digested MOF-808 sample.

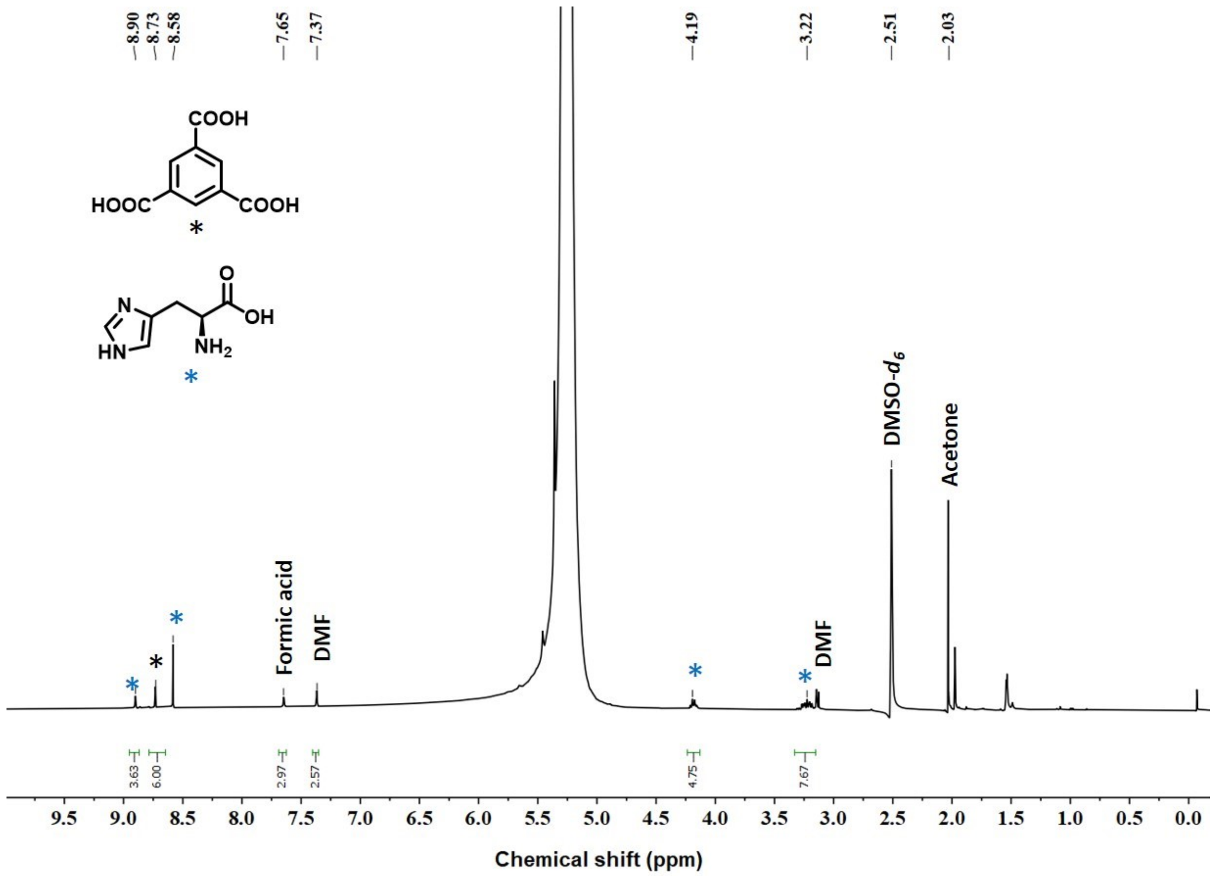

Figure S6: ^{1}H NMR spectrum of digested MOF-808-His sample.

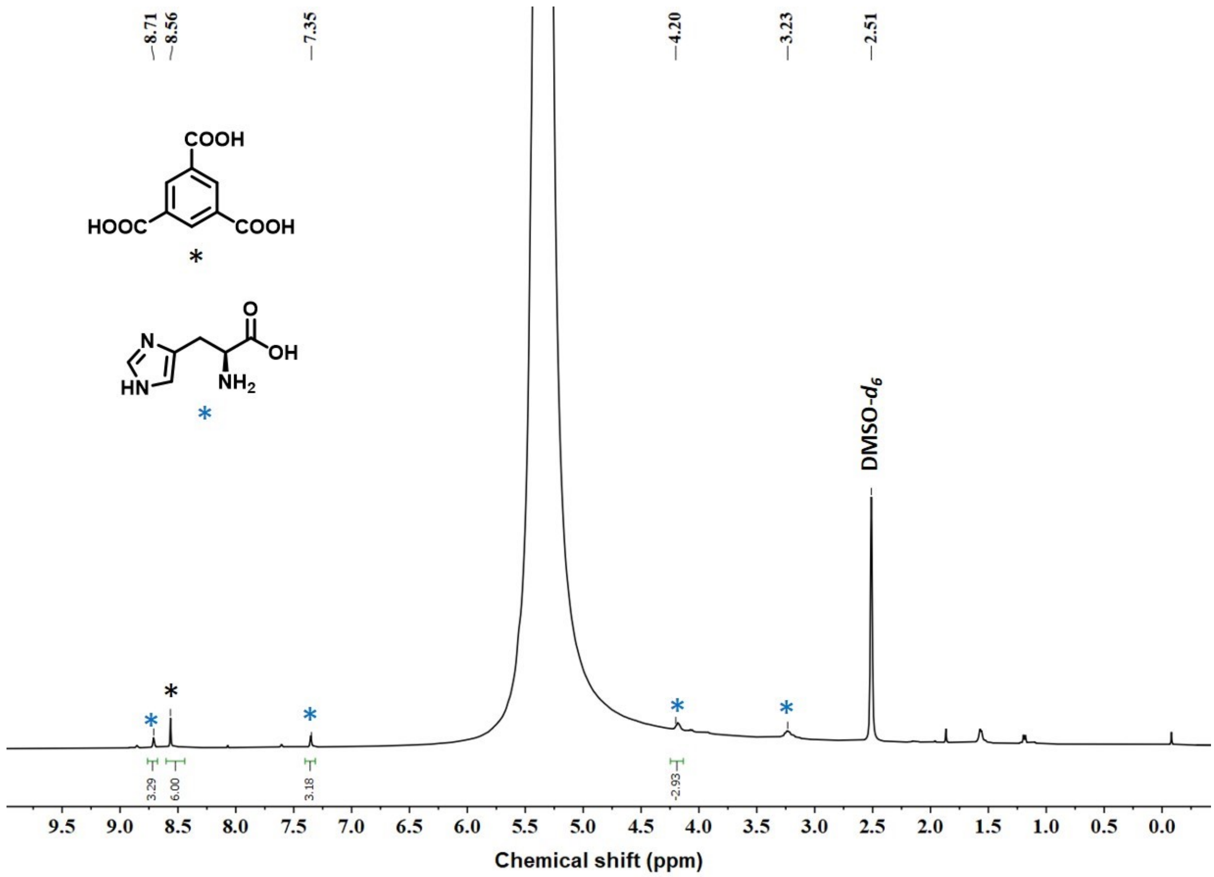

Figure S7: ^{1}H NMR spectrum of digested MOF-808-His-Cu sample.

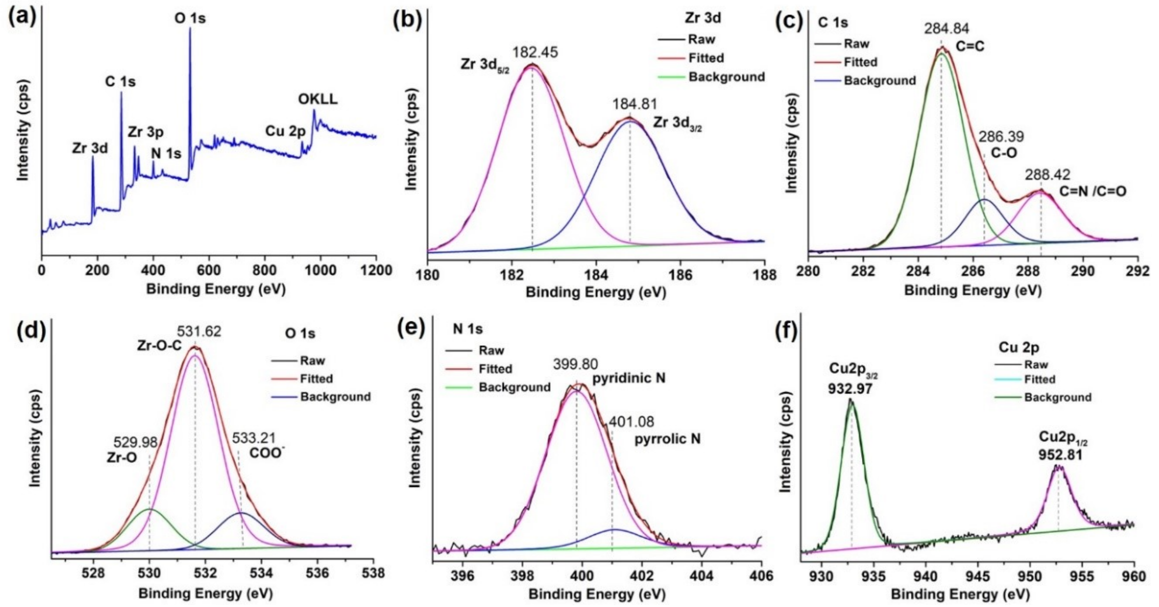

Figure S8: (a) XPS survey spectrum of MOF-808-His-Cu. Deconvoluted XPS spectrum of **(b)** Zr 3d **(c)** C 1s **(d)** O 1s **(e)** N 1s **(f)** Cu 2p in MOF-808-His-Cu. The N 1s high-resolution spectra reveal the presence of pyridinic (399.80 eV) and pyrrolic nitrogen (401.08 eV), derived from L-histidine.[16]

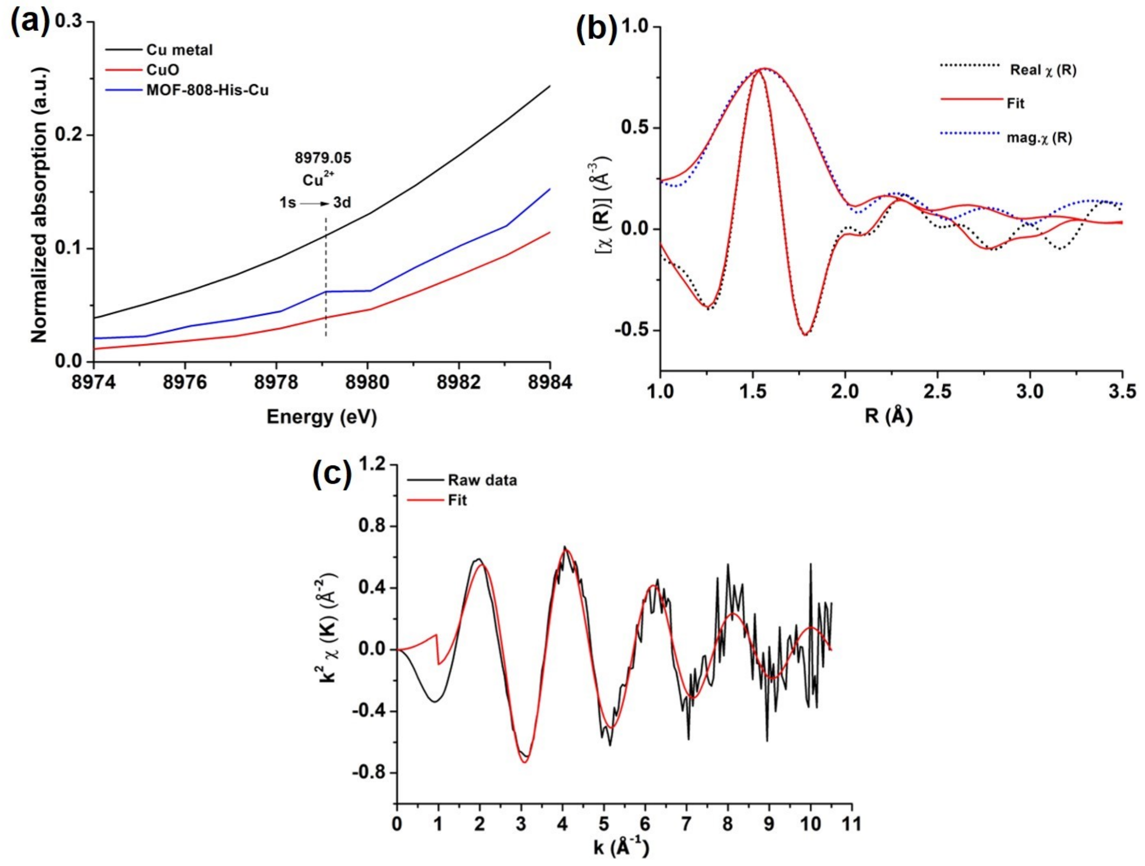

Figure S9: (a) Cu K-edge XANES spectrum displaying a weak peak near 8979.05 eV attributed to the dipole forbidden Cu^{2+} 1s→3d electronic transition, which is identified as the fingerprint of Cu^{2+}.[14] **(b)** Cu K-edge Fourier-transformed EXAFS spectra representing the magnitude (blue dots) and real components (black dots) of MOF-808-His-Cu. The data is fitted (red lines) in the range 3 – 11 $Å^{-1}$ in k -space and, 1 – 3 Å in R–space) **(c)** *k* -weighted Cu-EXAFS raw data (black line) of MOF-808-His-Cu with the best fit (red line) analyzed in the range $3 < k < 11$ $Å^{-1}$

Table S1: EXAFS fitting result of MOF-808-His-Cu.

**Ab-Sc pair**[a]	**N**[b]	**R**[c]	**DWF**[d]	**R-factor**[e]
Cu – N/(O)	2.74 ± 0.28	2.0021 ± 0.0045	0.0027 ± 0.0005	0.012
Cu – Cu	1.0 (kept fixed)	2.524 ± 0.0607	0.028 ± 0.01	

[a] Ab=absorber; Sc=scatterer. [b] Coordination number, [c] Distance (Å), [d] Debye-Waller factor ($Å^2$), [e]A measure of mean square sum of the misfit at each data point. Fit range: $3 < k < 11$ $Å^{-1}$; $1 < R < 3$ Å.

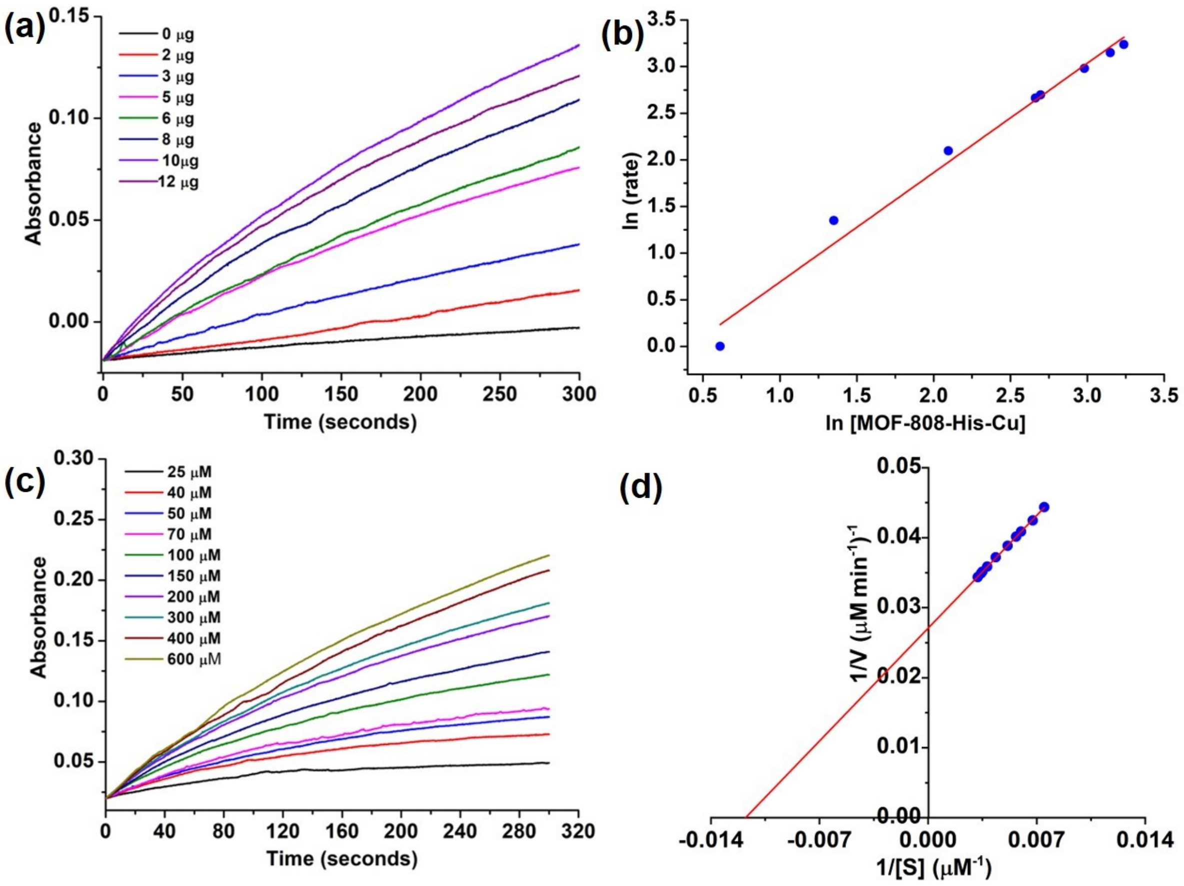

Figure S10: (a) Time-based absorbance spectra monitored at 415 nm, displays a proportional increase in absorbance of DTBC (200 μM) with the increasing concentrations of MOF-808-His-Cu (2-12 μg mL^{-1}) in PB (7.4) at 25 ^{o}C. **(b)** Plot of ln (rate) *versus* ln (MOF-808-His-Cu) revealing the first-order kinetics. **(c)** The line plot of absorbance at different concentrations of DTBC (25-600 μM), in presence of MOF-808-His-Cu (12 μg mL^{-1}) at pH 7.4. **(d)** Lineweaver Burk plot of the variation of concentration of DTBC in presence of MOF-808-His-Cu

Table S2. Comparison of catalytic parameters for reported oxidase nanozymes with that of MOF-808-His-Cu towards catechol substrate.

Material	Substrate	K_M (μM)	K_{cat} (s^{-1})	K_{cat}/K_M (M^{-1} s^{-1})	Reference
MOF-818	DTBC	810	0.383	473	[17]
CeO_2	DTBC	1262	6.28 x 10^{-4}	0.498	[17]
Pt NPs	DTBC	1811	0.0184	10.1	[17]
CeO_2@L-Phe	L-dopa	0.431	4.16x 10^{-13}	0.00965	[18]
CeO_2@D-Phe	L-dopa	0.168	3.12 x 10^{-13}	0.0186	[18]
Pt NPs	quercetin	54.37	244.82	-	[19]
MOF-808-His-Cu	DTBC	85.07	0.0476	550	This work

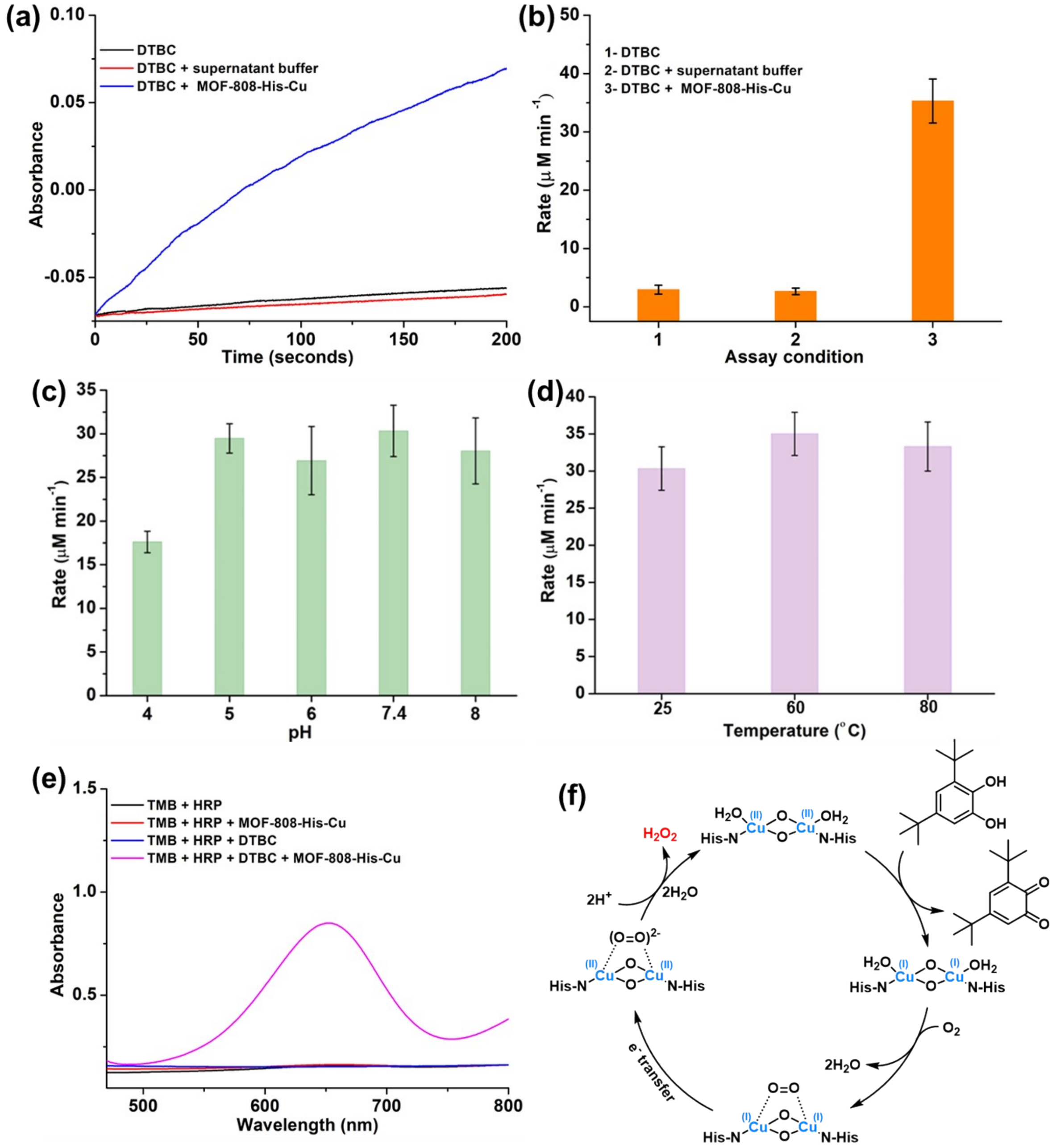

Figure S11: (a) Absorbance *versus* time plot exhibiting no oxidase activity of the supernatant due to leached Cu^{2+} ions. **(b)** The corresponding initial rate of the oxidase activity of the supernatant after incubating MOF-808-His-Cu in PB (7.4) at 25 °C. **(c and d)** Oxidase activity of MOF-808-His-Cu using DTBC at pH 7.4, after treatment of MOF-808-His-Cu under different pH conditions (4, 5, 6, 7.4, and, 8) and temperatures (25 °C, 60 °C and 80 °C), respectively. **(e)** Detection of formed H_2O_2 during DTBC oxidation by MOF-808-His-Cu using TMB-HRP assay. **(f)** Proposed catalytic cycle catechol oxidase-like activity of MOF-808-His-Cu showing H_2O_2 generation.

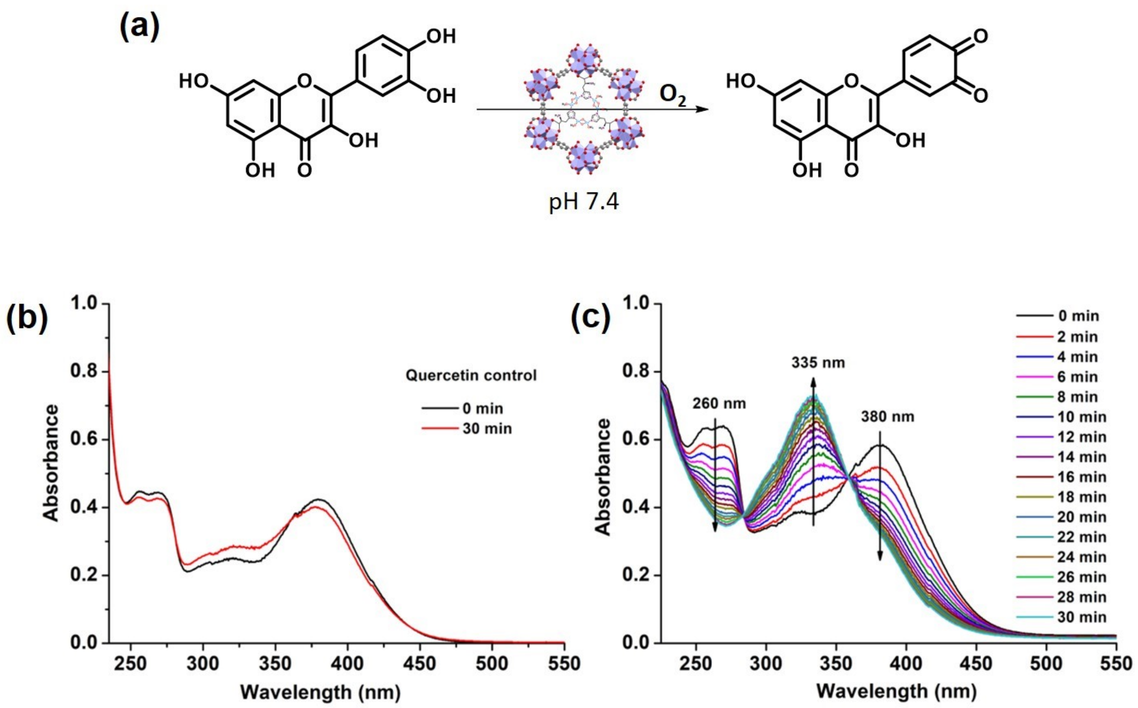

Figure S12:(a) Schematic illustration of oxidase-like activity of MOF-808-His-Cu using quercetin as a catechol substrate. UV-vis absorption spectra of **(b)** quercetin (30 μM) control and, **(c)** quercetin (30 μM) oxidation catalyzed by MOF-808-His-Cu (12 μg mL^{-1}) at pH 7.4 (50 mM PB) at 25 ^{o}C.

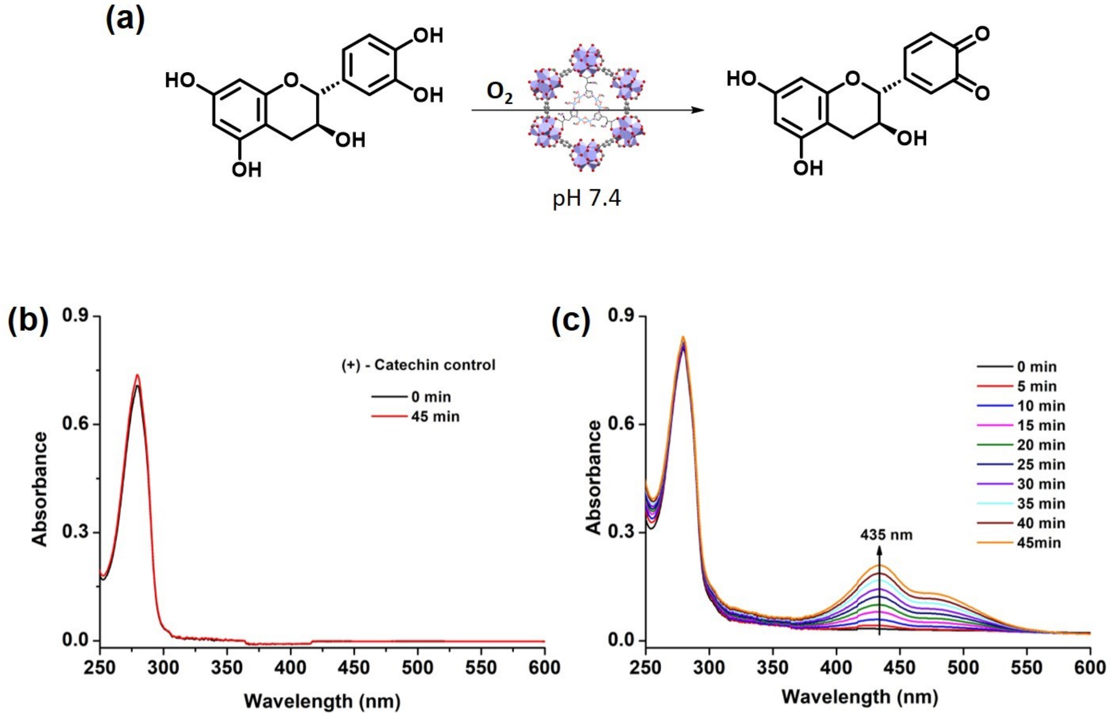

Figure S13:(a) Schematic illustration of oxidase-like activity of MOF-808-His-Cu using (+)-catechin as catechol substrate. UV-vis absorption spectra of **(b)** (+)-catechin (200

μM) control and, **(c)** (+)-catechin (200μM) oxidation catalyzed by MOF-808-His-Cu (12 μg mL^{-1}) at pH 7.4 (50 mM PB), 25 ^{o}C.

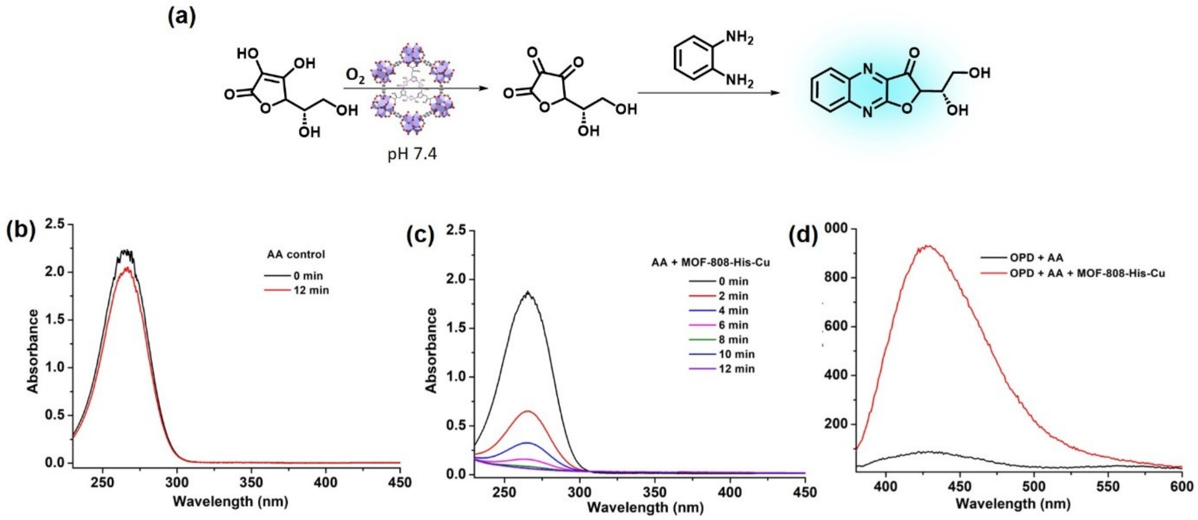

Figure S14: (a) Schematic representation of AA oxidase-mimetic activity of MOF-808-His-Cu and, detecting the formation of DHA using OPD as a fluorescence-based probe. **(b)** UV-vis absorption spectra of AA (125 μM) and, **(c)** AA (125 μM) in the presence of MOF-808-His-Cu (12 μg mL^{-1}) at pH 7.4, 25 ^{o}C **(d)** Fluorescence emission spectra of 3-(1,2-dihydroxyethyl)furo-[3,4-b]quinoxaline-1-one exhibiting intense fluorescence at 425 nm (λ_{ex} = 350 nm) and its control.

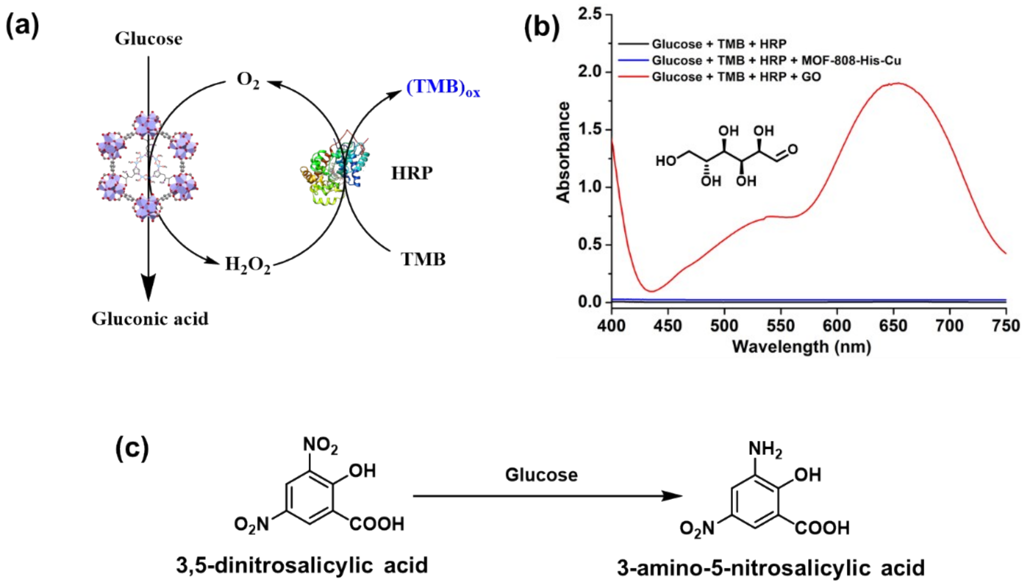

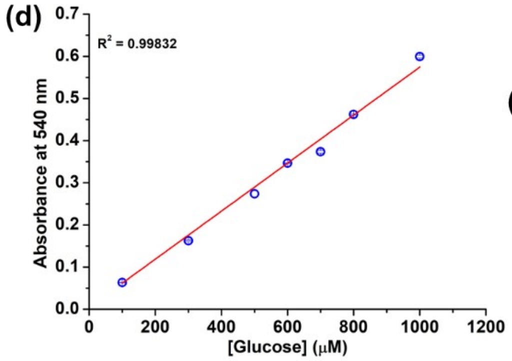

(e)

Assay conditions	A_{540nm}	[Glucose] (μM)
Glucose control	0.326 $\pm$ 0.008	565.5
Glucose + MOF-808-His-Cu	0.327 $\pm$ 0.008	566.1

Figure S15: (a) Evaluation of glucose oxidation in the presence of MOF-808-His-Cu and using HRP/TMB to probe H_2O_2 at pH 7.4. **(b)** UV-vis absorption spectra of glucose (1 M) catalyzed by MOF-808-His-Cu (12 μg mL^{-1}) in the presence of HRP (0.1 μg mL^{-1}), TMB (0.4 mM) at pH 7.4, 25 ^oC. As a positive control, the reaction was performed in the presence of 1 U mL^{-1} of GO. **(c)** Conversion of 3,5-dinitrosalicylic acid (DNS) to 3-amino-5-nitrosalicylic acid in the presence of glucose. This assay is used for the detection and quantification of glucose. **(d)** A standard calibration curve for estimation of glucose (0-1000μM) using DNS assay. **(e)** Tabular data showing the absorbance value at 540 nm and the corresponding glucose concentration calculated from the calibration plot.

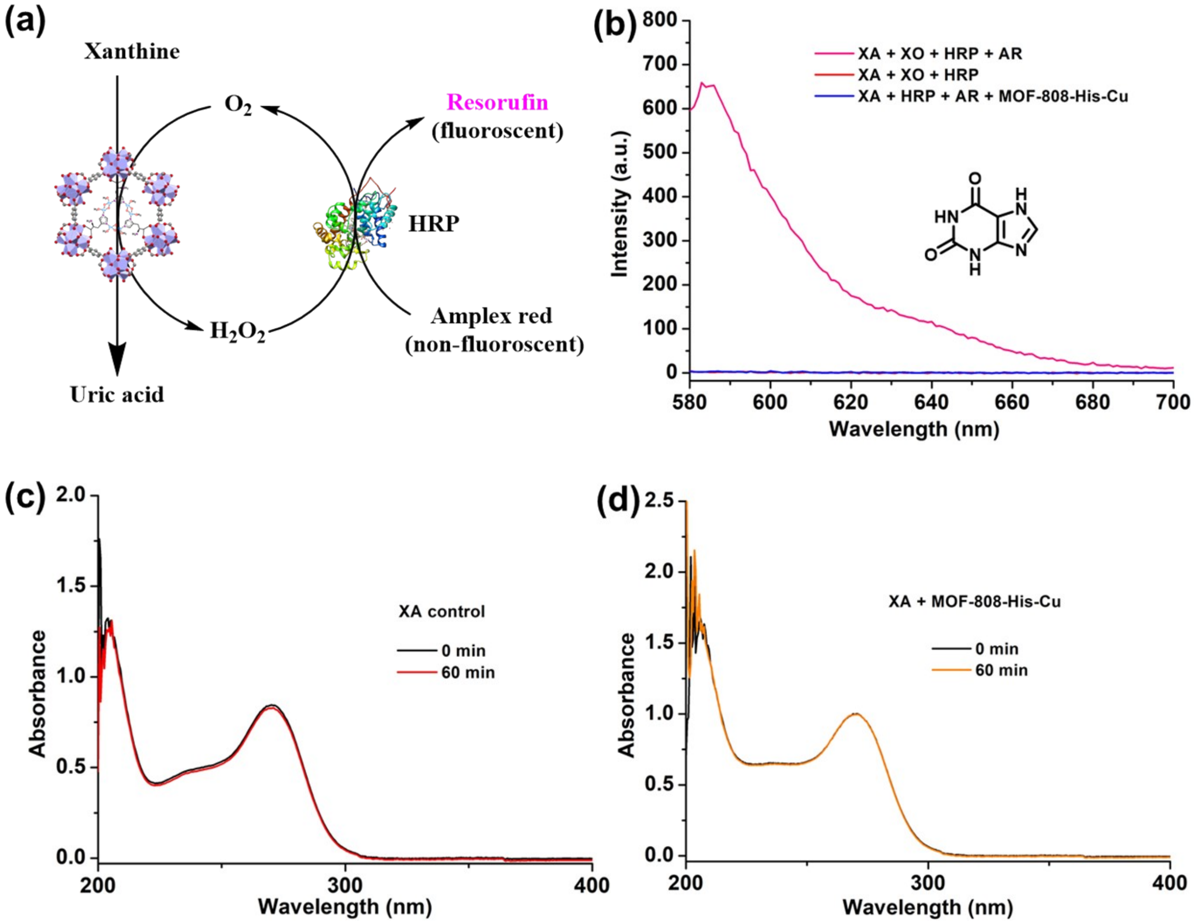

Figure S16: (a) Evaluation of XA oxidation in the presence of MOF-808-His-Cu and employing AR/HRP assay for the detection of H_2O_2. **(b)** Fluorescence emission spectra of XA (500 μM) catalyzed by MOF-808-His-Cu (12 μg mL^{-1}) in the presence of AR (5 μM), and HRP (2.5 U mL^{-1}) in PB (7.4) (λ_{ex} = 550 nm). As a positive control, the reaction was performed in the presence of XO (16.8 mU mL^{-1}). **(c-d)** Evaluation of consumption of XA in the absence and presence of MOF-808-His-Cu as studied by monitoring the peak at 270 nm in the UV-vis spectra, respectively.

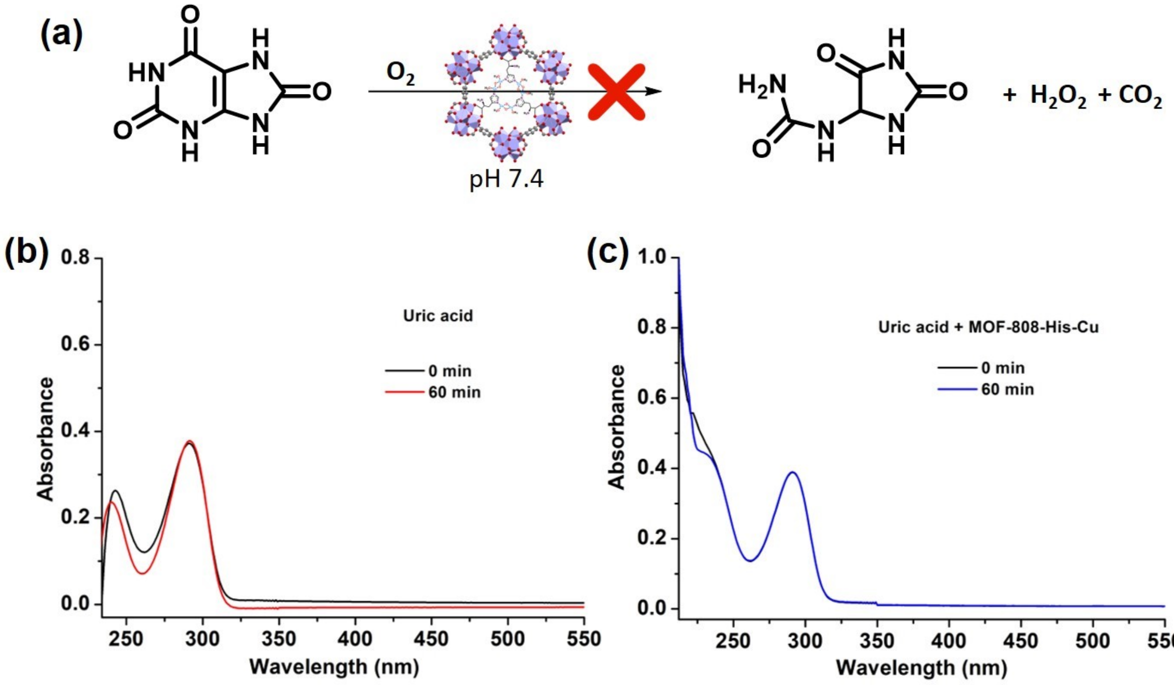

Figure S17: **(a)** Evaluation of UA oxidation in presence of MOF-808-His-Cu. UV-vis absorption spectra of **(b)** UA (50 μM) control and, **(c)** UA (50 μM) in presence of 12 μg mL^{-1} of MOF-808-His-Cu in PB (7.4) after incubating for 60 min at 25 ^{o}C (monitored at λ_{max} 290 nm).

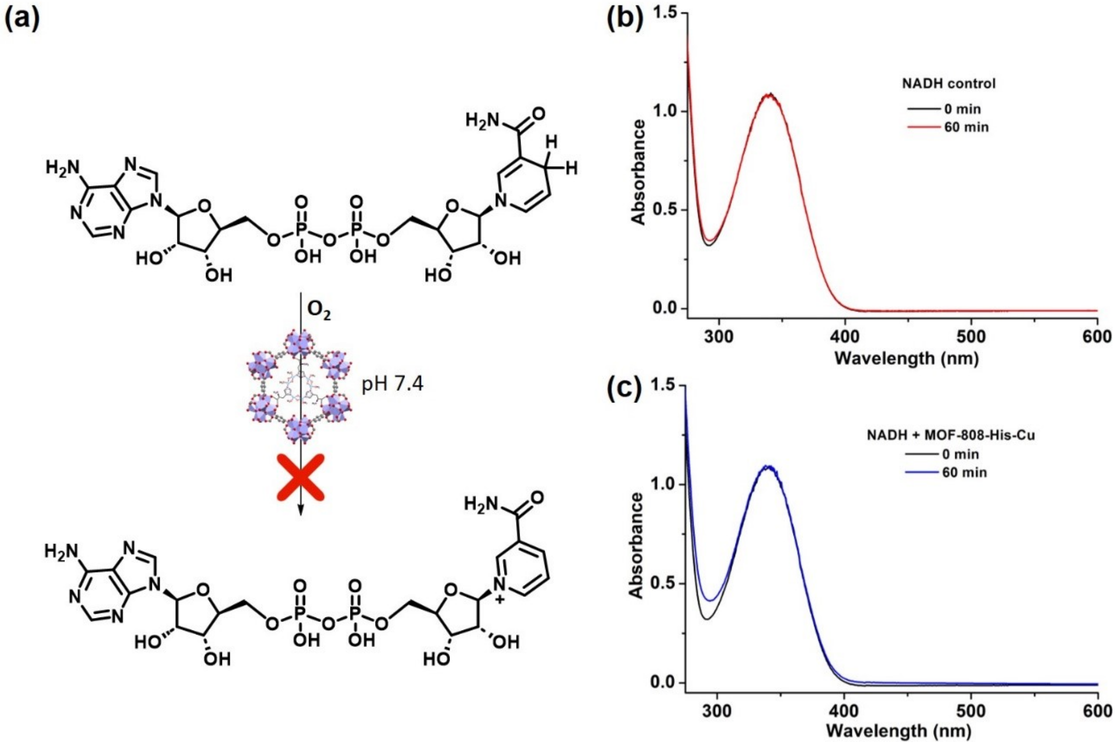

Figure S18: **(a)** Evaluation of NADH oxidation in presence of MOF-808-His-Cu. UV-vis absorption spectra of **(b)** NADH (0.2 mM) control and, **(c)** NADH (0.2 mM) in presence of 12 μg mL^{-1} of MOF-808-His-Cu in PB (7.4) after incubating for 60 min at 25 ^{o}C (monitored at λ_{max} 340 nm).

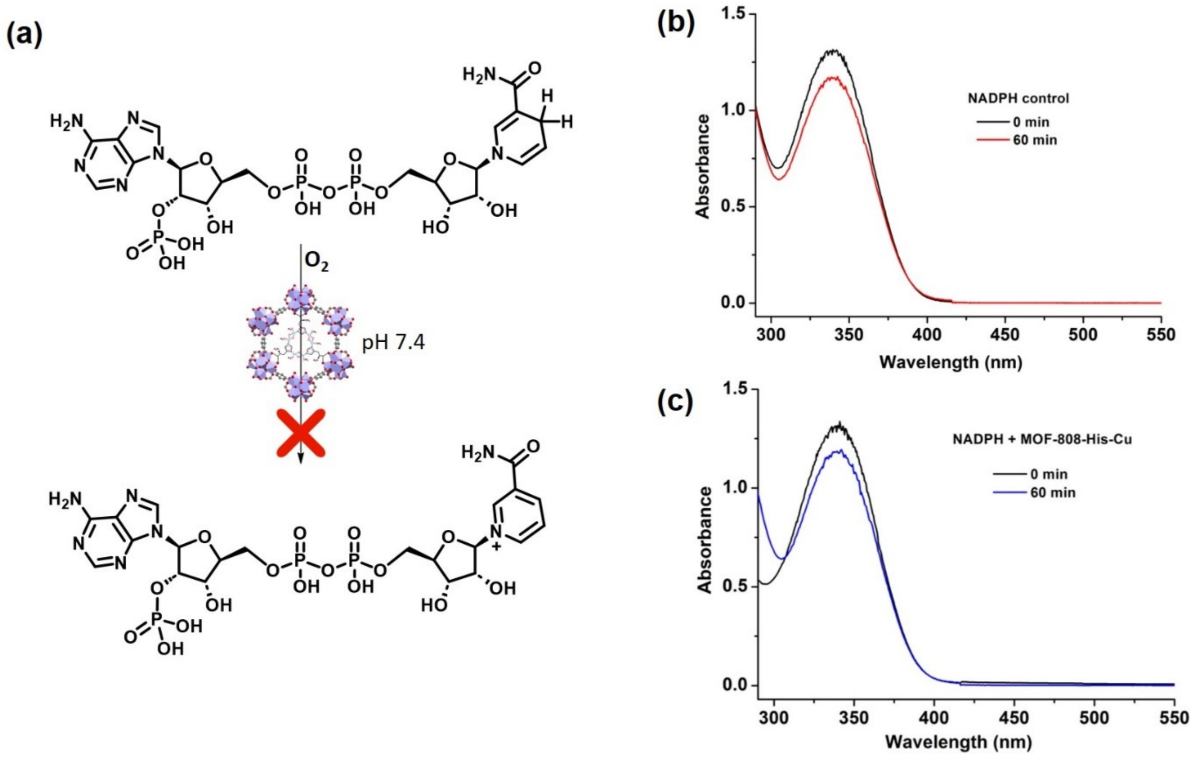

Figure S19: (a) Evaluation of NADPH oxidation in presence of MOF-808-His-Cu. UV-vis absorption spectra of **(b)** NADPH (0.2 mM) control and, **(c)** NADPH (0.2 mM) in presence of 12 µg mL^{-1} of MOF-808-His-Cu in PB (7.4) after incubating for 60 min at 25 ^{o}C (monitored at λ_{max} 340 nm).

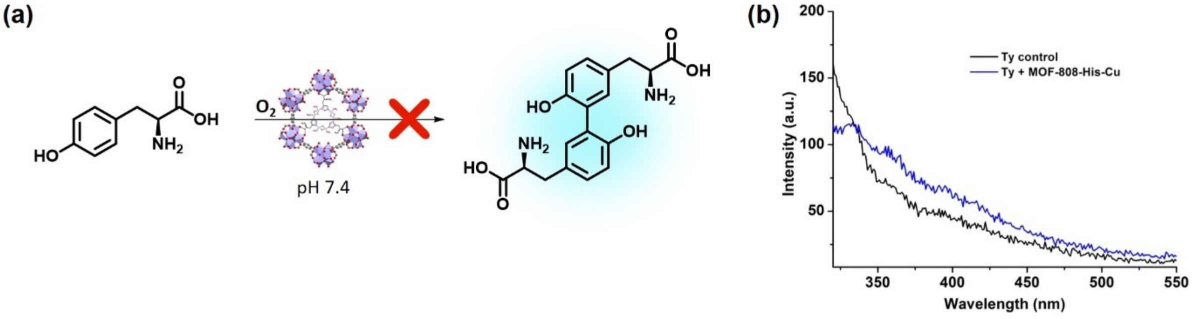

Figure S20: (a) Evaluation of oxidation of Ty to dityrosine in the presence of MOF-808-His-Cu. **(b)** Fluorescence emission spectra of Ty (500 µM) after incubation with 12 µg mL^{-1} of MOF-808-His-Cu in PB (7.4) for 60 min at 25 ^{o}C (λ_{ex} 300 nm) and its control.

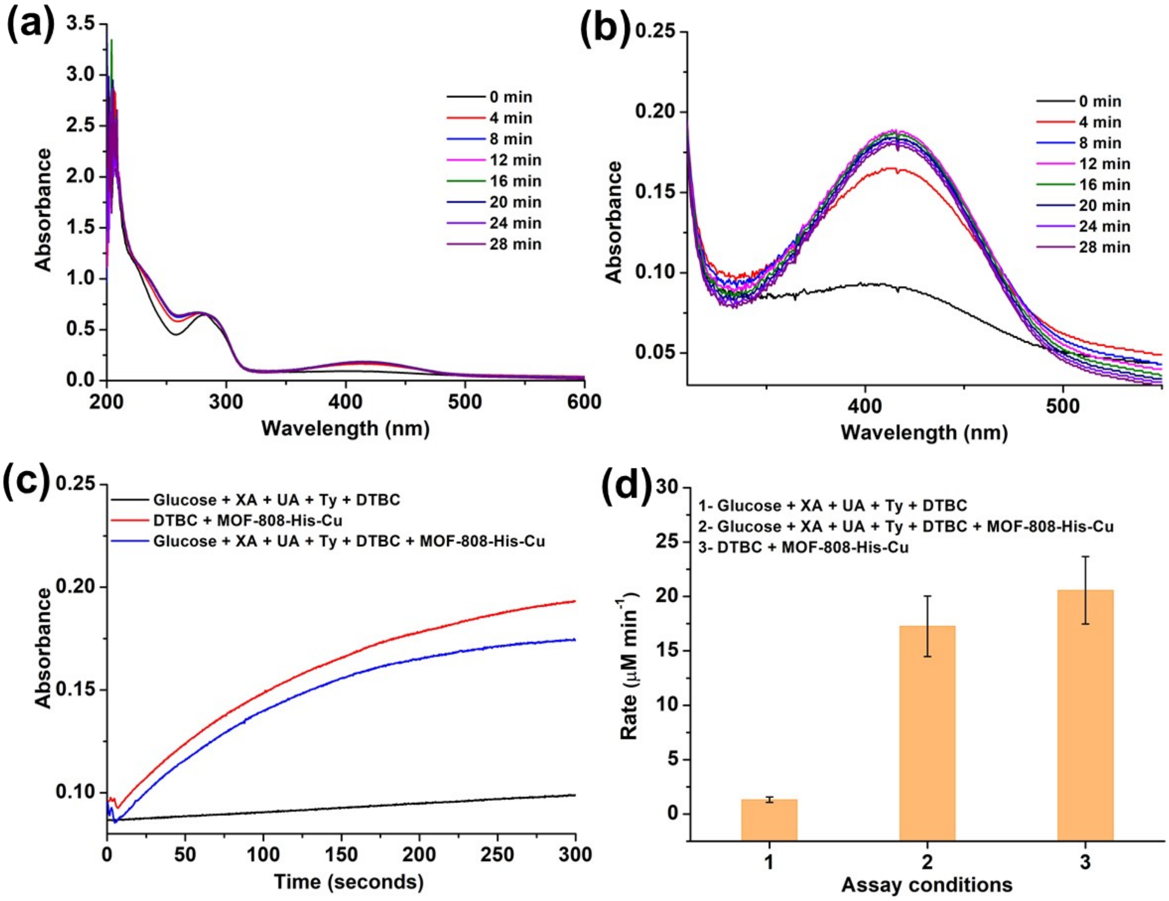

Figure S21: (a) Evaluation of substrate selectivity of MOF-808-His-Cu in the presence of a mixture containing 100 μM of DTBC, glucose, XA, Ty, and, UA monitored for 28 min. **(b)** Expanded view of spectra presented in **(a)**. **(c)** Evaluation of the formation of DTBQ by time-drive mode UV-vis spectroscopy during the oxidation by MOF-808-His-Cu in the presence of the aforementioned mixture of substrates. **(d)** Initial rates of the reactions are presented in plot **(c).**

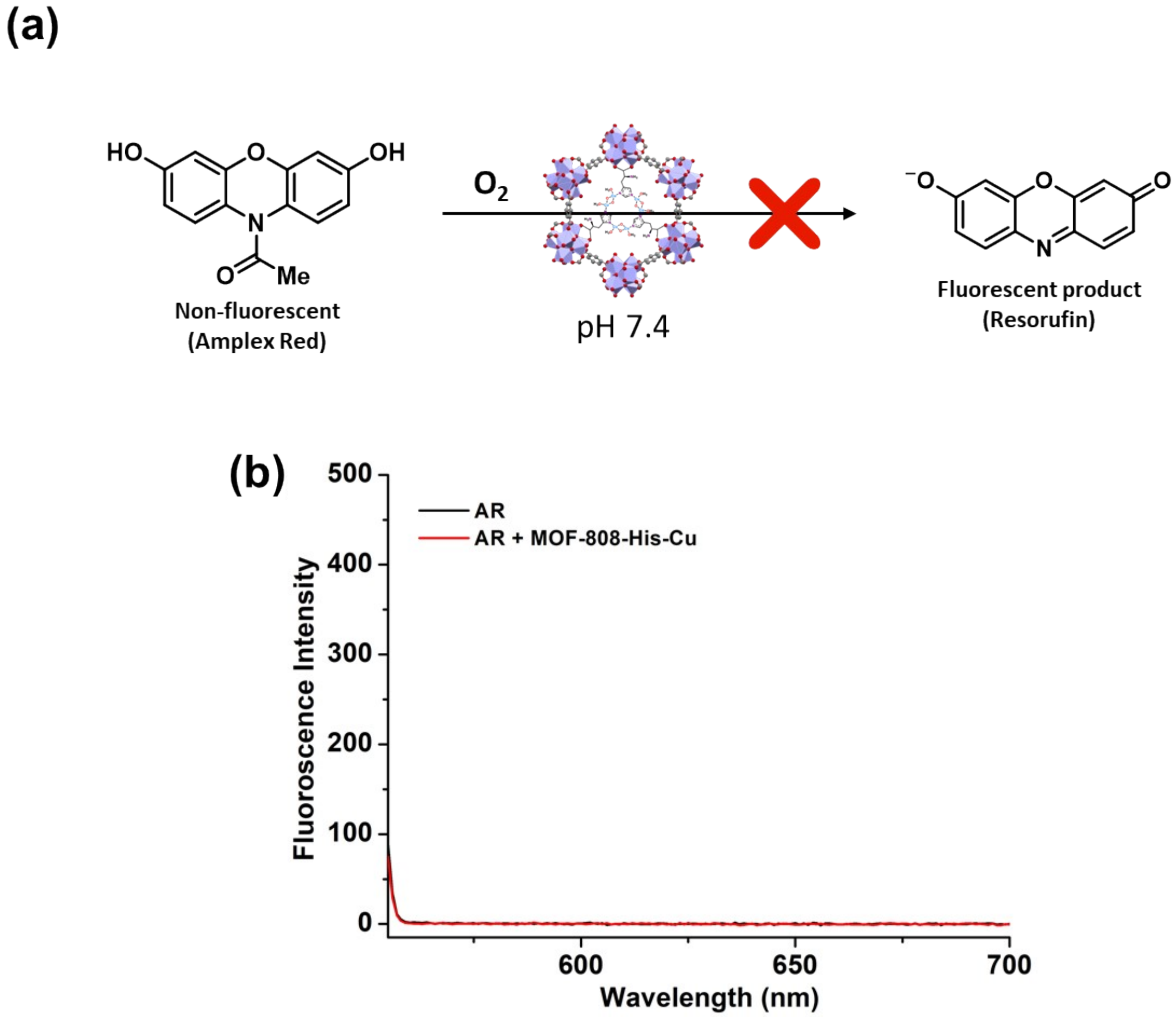

Figure S22: (a) Evaluation of the oxidase-like activity of MOF-808-His-Cu using AR at pH 7.4. **(b)** Fluorescence spectra of AR (1 μM) in presence of MOF-808-His-Cu (12 μg mL^{-1}) in PB (7.4), 25 ^{o}C after incubation for 30 min (λ_{ex} = 550 nm/ λ_{em} = 583 nm).

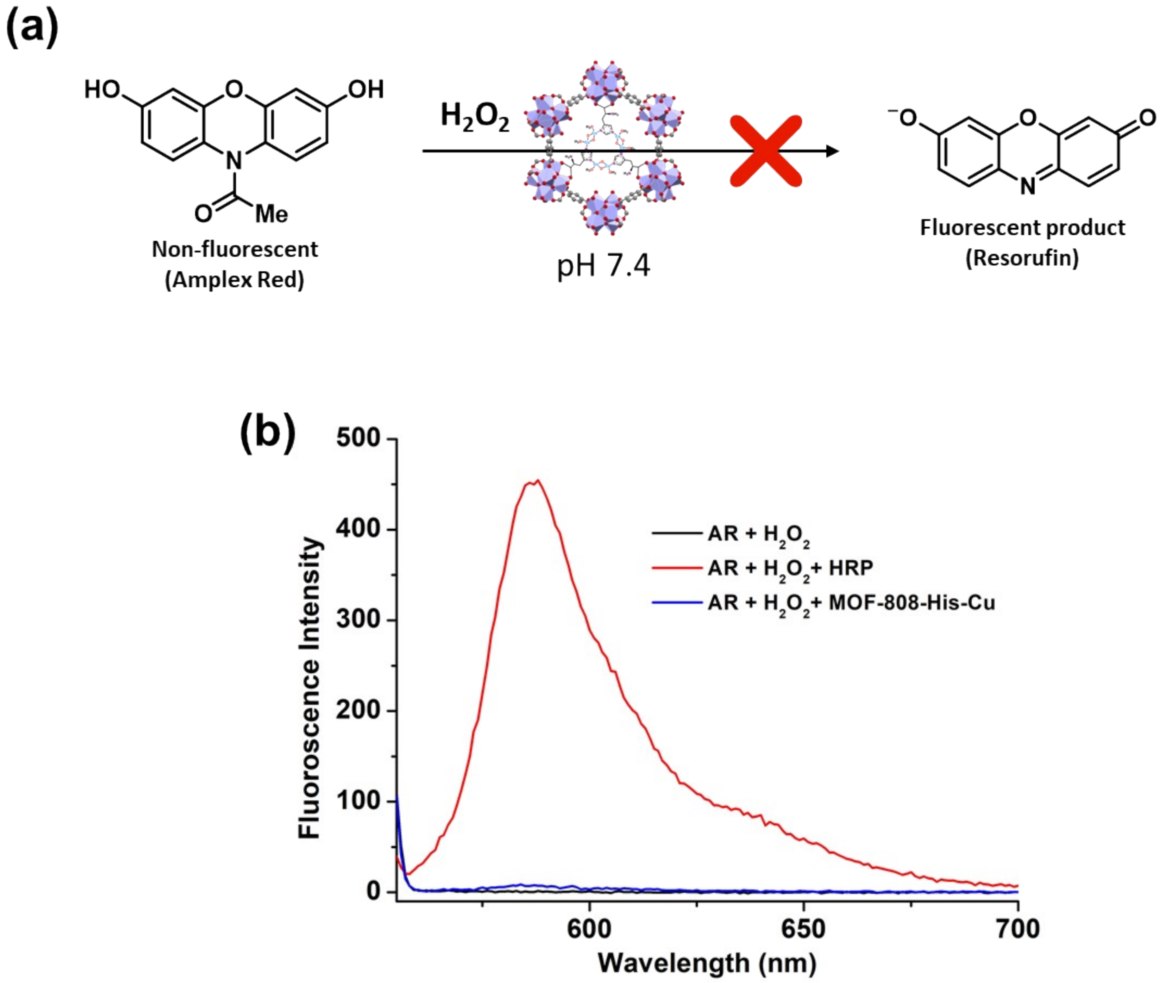

Figure S23: (a) Evaluation of the peroxidase-like activity of MOF-808-His-Cu using AR at pH 7.4. **(b)** Fluorescence spectra of AR (1 µM) in the presence of 1 mM H_2O_2 after incubation with 12 µg mL^{-1} of MOF-808-His-Cu at 7.4, 25 ^oC for 30 min (λ_{ex} = 550 nm/ λ_{em} = 583 nm).

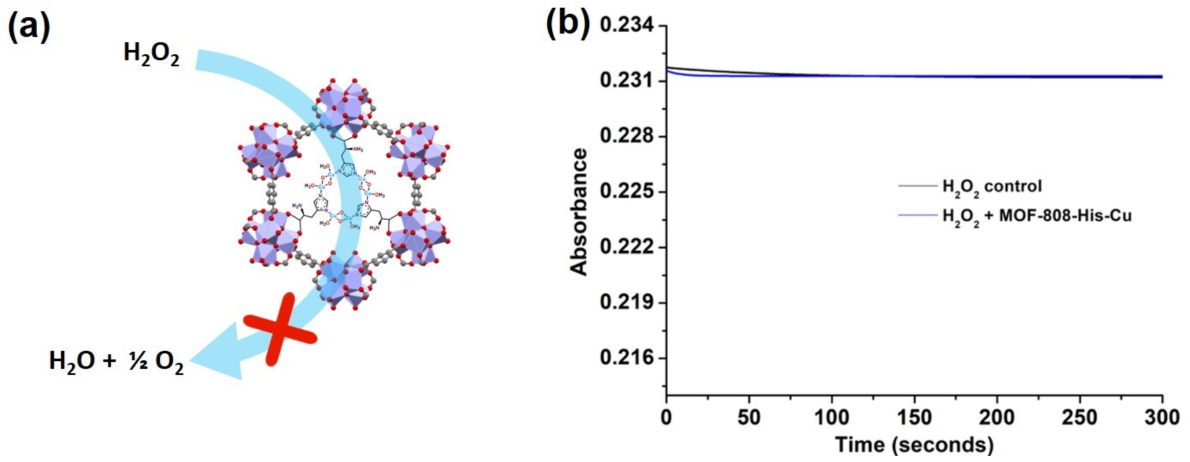

Figure S24: (a) To determine the catalase-like activity of MOF-808-His-Cu at pH 7.4. **(b)** Time-dependent absorption spectra of 5 mM H_2O_2 in the presence of 12 µg mL^{-1} of MOF-808-His-Cu at 7.4, 25 ^oC for 300 seconds monitored at 240 nm in kinetics mode and its control.

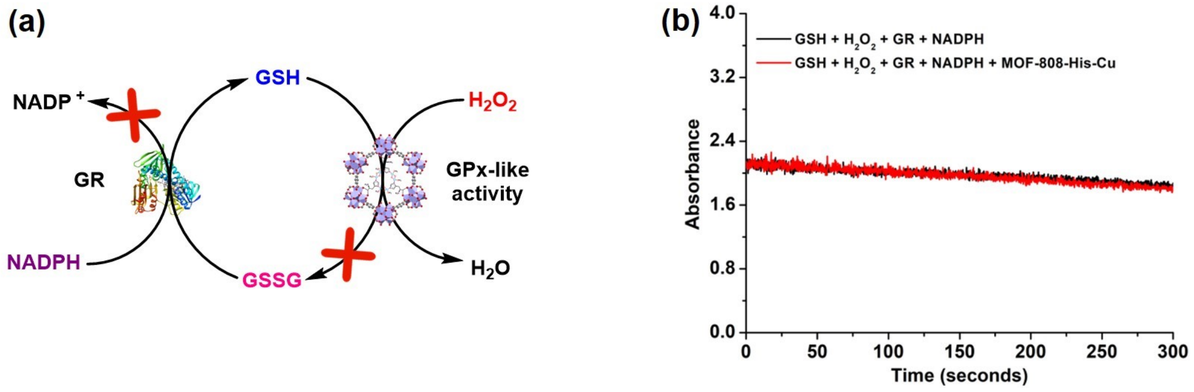

Figure S25: (a) Schematic diagram depicting the classical GRx-coupled assay for evaluating the GPx-like activity of MOF-808-His-Cu at pH 7.4. **(b)** Time-dependent absorption spectra of MOF-808-His-Cu (12 µg mL^{-1}), GSH (2 mM), NADPH (0.4 mM), GR (1.7 U mL^{-1}), H_2O_2 (240 µM) in PB (pH 7.4), monitored at 340 nm and its control.

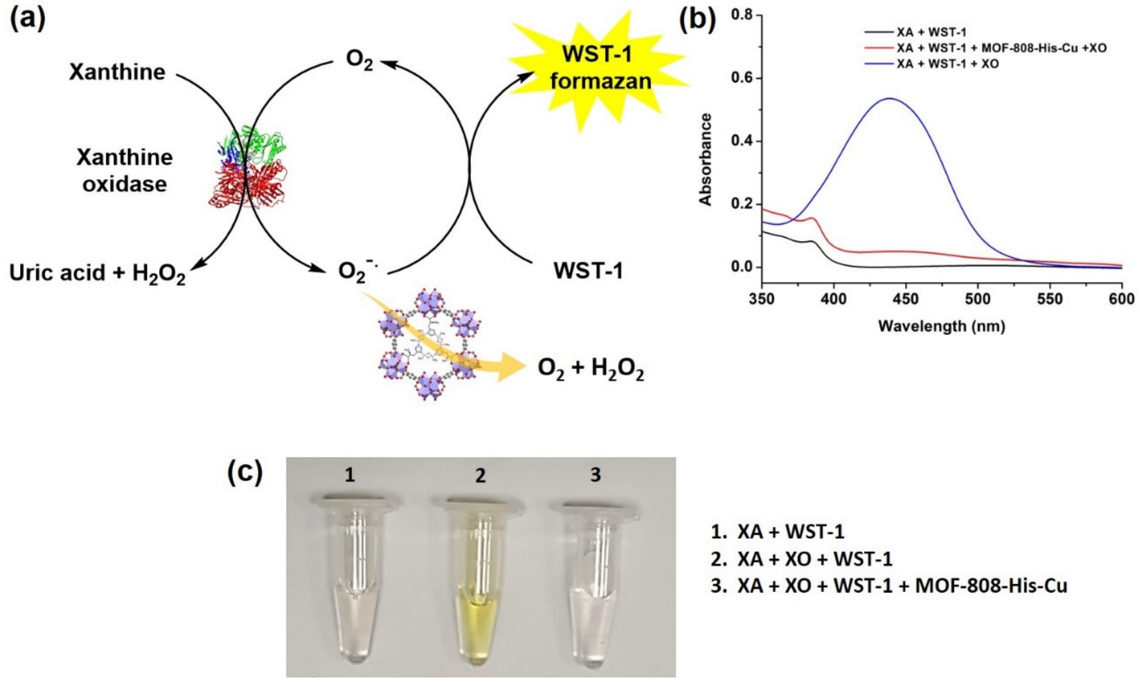

Figure S26: (a) Schematic representation of the $O_2^{-\bullet}$ detection assay using WST-1 dye to evaluate the SOD like-activity in presence of MOF-808-His-Cu at pH 7.4. **(b)** UV-vis absorption spectra of XA (500 µM) with XO (33.6 mU mL^{-1}) in presence of 12 µg mL^{-1} MOF-808-His-Cu at 7.4, 25 ^{o}C. The control reactions in the absence of MOF-808-His-Cu were also performed. **(c)** The reaction of XA, XO, with MOF-808-His-Cu in presence of WST-1 dye did not display yellow color confirming the SOD-like activity of MOF-808-His-Cu.

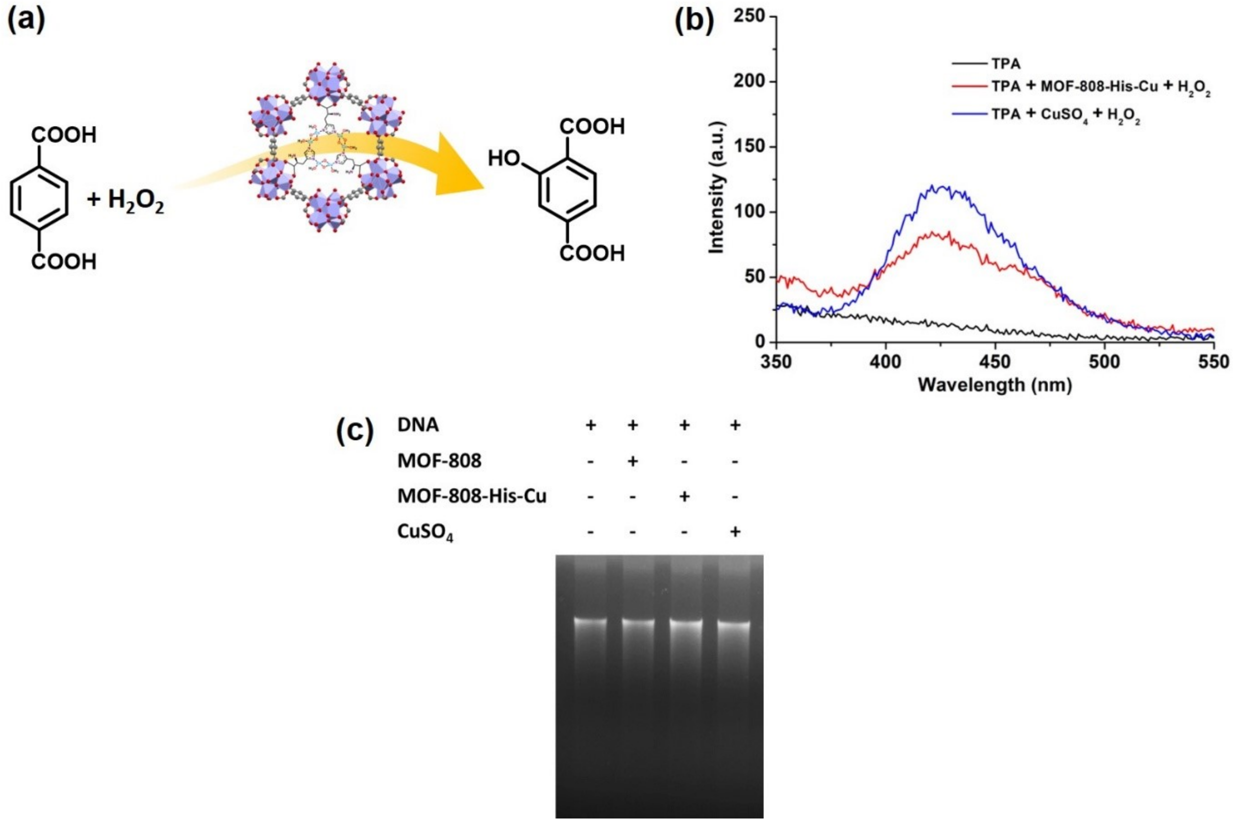

Figure S27: (a) Schematic illustration depicting the Fenton reaction of MOF-808-His-Cu probed by TPA. **(b)** Fluorescence emission spectra of TPA (0.5 mM) in the presence of 1 mM H_2O_2 catalyzed by MOF-808-His-Cu (50 μgmL^{-1}), (λ_{ex} = 315 nm). As a positive control, the reaction was performed with an equivalent amount of $CuSO_4$ under similar reaction conditions. **(c)** Assessment of *in vitro* DNA damage (Calf Thymus type 1 DNA) in the absence of H_2O_2 for various catalysts at pH 7.4, 25 ^{o}C.

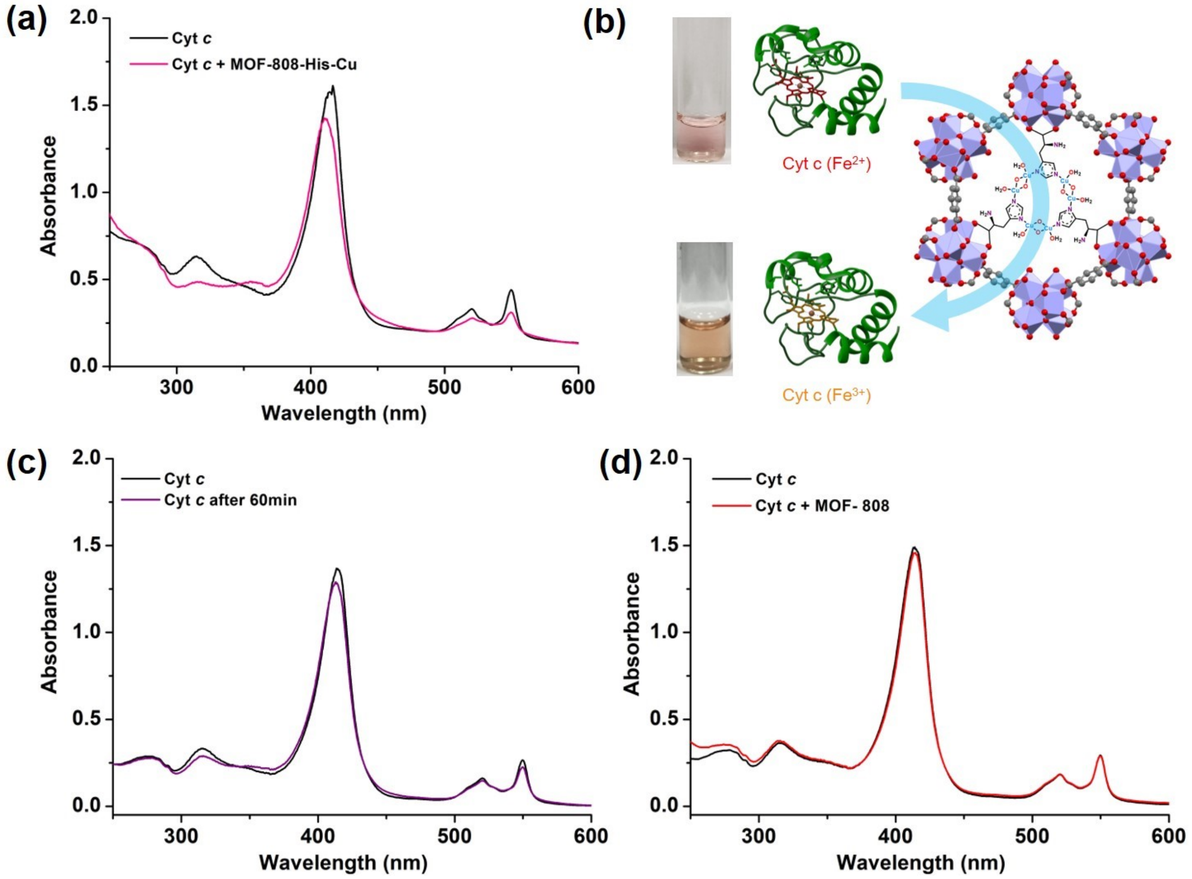

Figure S28: (a) The COX of MOF-808-His-Cu was monitored by UV-vis spectrophotometer for the reaction mixture containing Cyt *c* (10μM) in PB (7.4) in the presence of 12 μg mL^{-1} of MOF-808-His-Cu at 25 ^{o}C. **(b)** The color change from pink to yellowish-orange on oxidation of reduced Cyt *c* (Fe^{2+}) to Cyt *c* (Fe^{3+}) in the presence of MOF-808-His-Cu. UV-vis absorption spectra of (**c**) Cyt *c* (10 μM) in PB (7.4) before and after 60 min. **(d)** Cyt *c* (10 μM) in PB (7.4) in the presence of MOF-808 (12 μg mL^{-1}) monitored for 60 min at 25 ^{o}C.

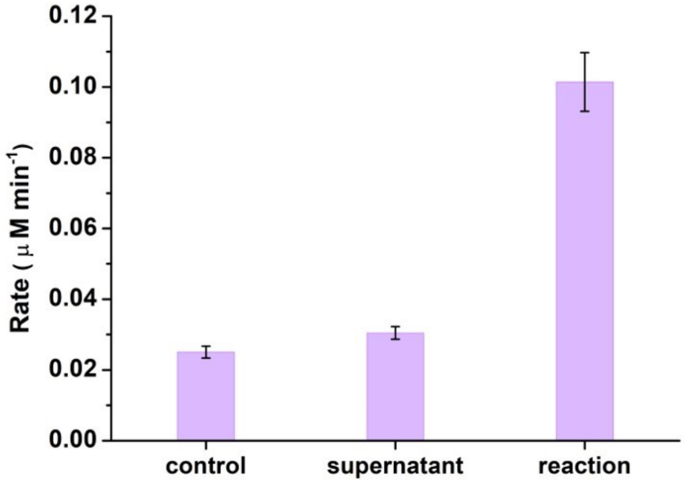

Figure S29: (a) The corresponding initial rate of the COX of supernatant after incubating MOF-808-His-Cu in PB (7.4) at 25 ^{o}C .

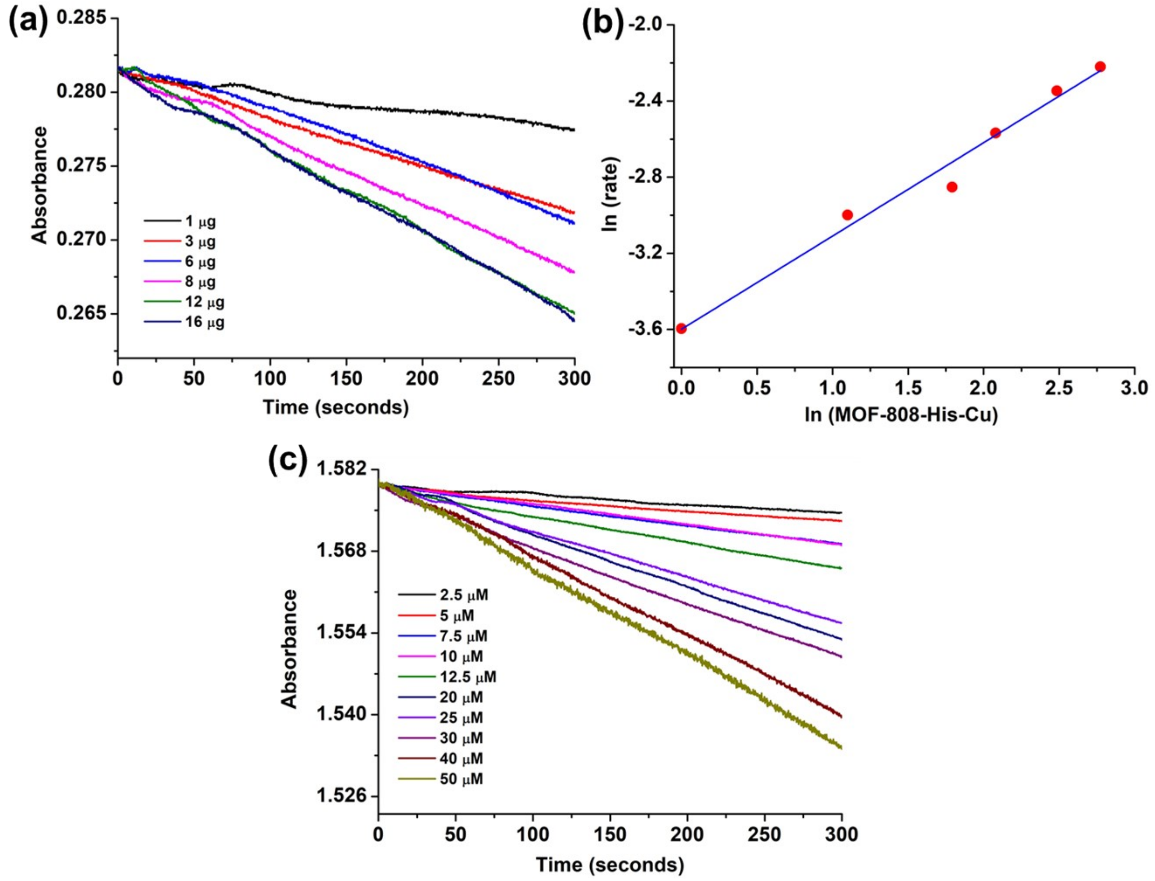

Figure S30: (a) Time-based absorbance spectra monitored at 550 nm, display a decrease in absorbance of reduced Cyt *c* (10 μM) with the increasing concentrations of MOF-808-His-Cu (1-16 μg mL^{-1}) in PB (7.4) at 25 ^{o}C. **(b)** Plot of ln (rate) *versus* ln [MOF-808-His-Cu]. **(c)** Time-based absorbance spectra for increasing concentration of reduced Cyt *c* (2.5 – 50 μM) in the presence of MOF-808-His-Cu (12 μg mL^{-1}) in PB (7.4) monitored at 550 nm.

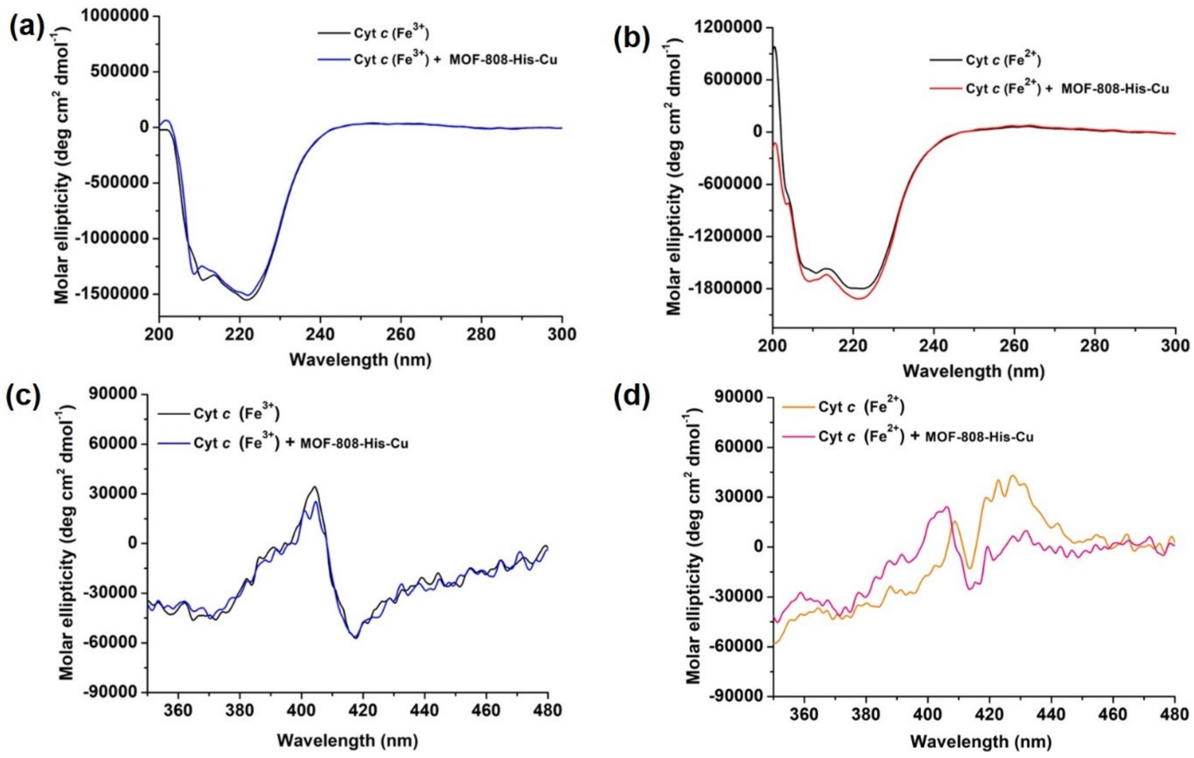

Figure S31: The CD spectra were recorded for 50 µM of reduced (Fe^{2+}) and oxidized (Fe^{3+}) Cyt *c* in the presence and absence of MOF-808-His-Cu (50 µg mL^{-1}). CD spectra in the wavelength range of 200-300 nm for **(a)** oxidized (Fe^{3+}) Cyt *c* and, **(b)** reduced (Fe^{2+}) Cyt *c* with and without MOF-808-His-Cu. CD spectra in the wavelength range of 300-500 nm for **(c)** oxidized (Fe^{3+}) Cyt *c* and, **(d)** reduced (Fe^{2+}) Cyt *c* with and without MOF-808-His-Cu.

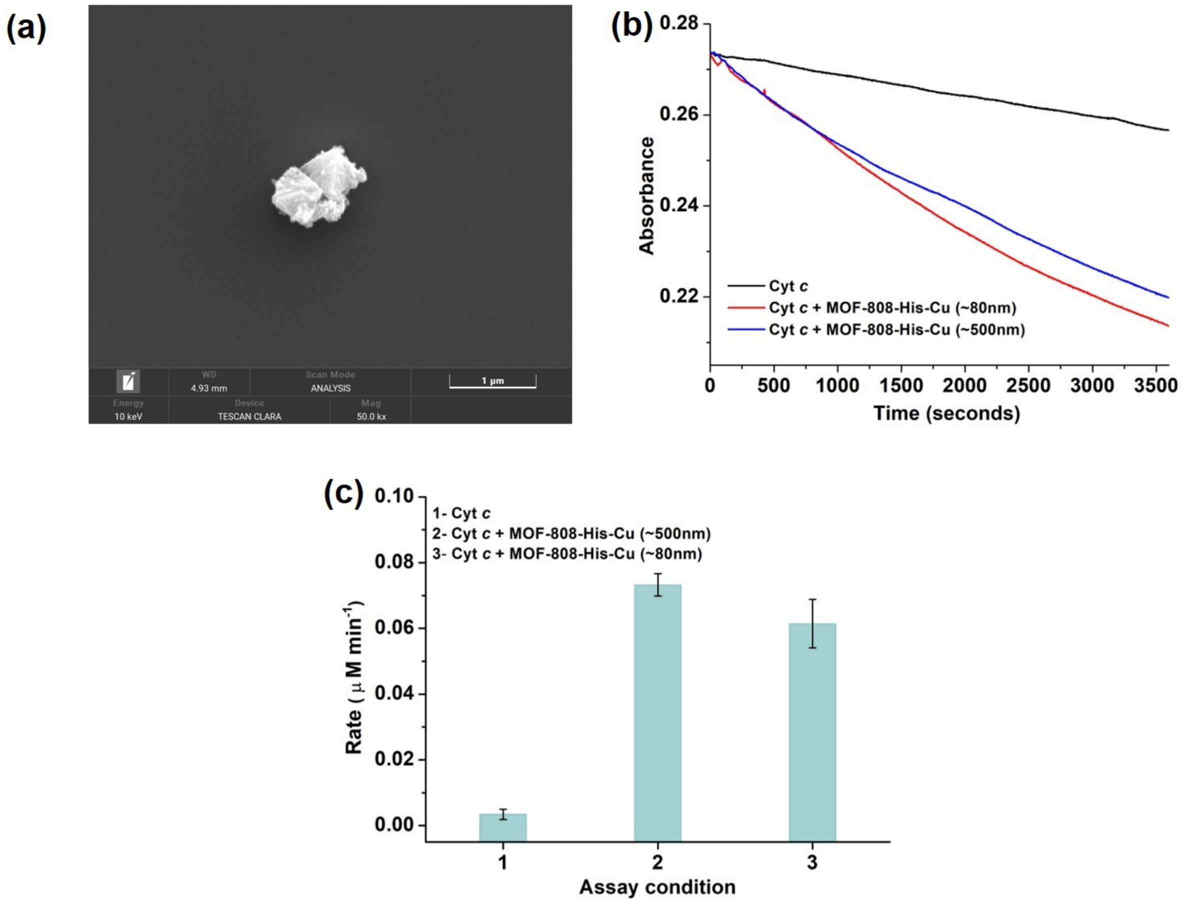

Figure S32: Effect of size of MOF-808-His-Cu on COX activity **(a)** SEM image of MOF-808-His-Cu (~500 nm size) (scale bar 1 µm). **(b)** Time-based absorbance spectra of Cyt *c* in the presence of 12 µg mL^{-1} MOF-808-His-Cu (~500 nm) at 550 nm, and **(c)** The corresponding bar diagram showing the initial rate of COX activity remained the same irrespective of the size of MOF-808-His-Cu.

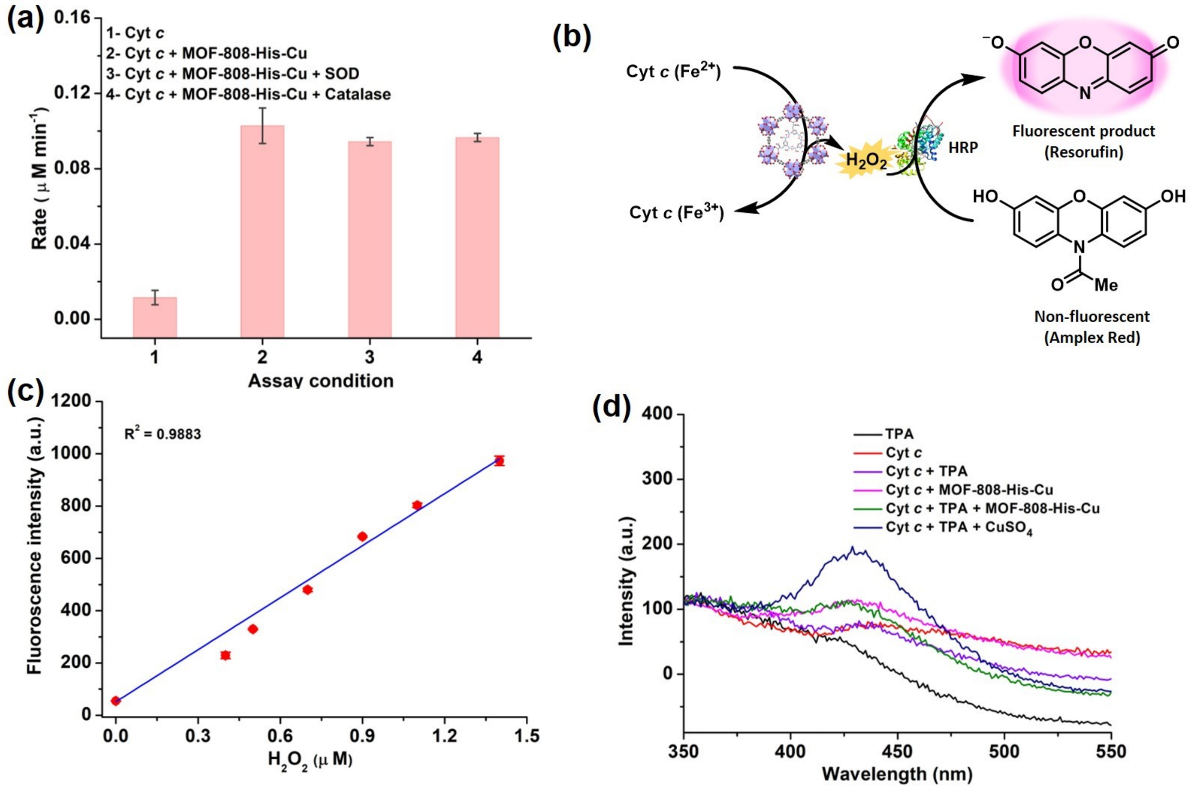

Figure S33: (a) The initial rate for Cyt *c* (10 μM) oxidation in presence of 12 μg mL^{-1} MOF-808-His-Cu along with the SOD and catalase enzyme in PB (7.4) at 25 ^oC. **(b)** Schematic diagram depicting AR/ HRP assay for the detection of H_2O_2 formed during Cyt *c* oxidation. **(c)** The calibration plot of AR/ HRP assay by varying the concentration of H_2O_2 (λ_{ex} = 550 nm/ λ_{em} = 583 nm). **(d)** Fluorescence emission spectra of TPA (0.5 mM) in presence of Cyt *c* and MOF-808-His-Cu for the detection of hydroxyl radical generated during Cyt *c* oxidation. (λ_{ex} = 315 nm).

References

1 H. Dong, G.-X. Yang, X. Zhang, X.-B. Meng, J.-L. Sheng, X.-J. Sun, Y.-J. Feng and F.-M. Zhang, *Chem. Eur. J.* 2018, **24**, 17148–17154.

2 J. Baek, B. Rungtaweevoranit, X. Pei, M. Park, S. C. Fakra, Y.-S. Liu, R. Matheu, S. A. Alshmimri, S. Alshehri, C. A. Trickett, G. A. Somorjai and O. M. Yaghi, *J. Am. Chem. Soc*. 2018, **140**, 18208–18216.

3 H. Lyu, O. I.-F. Chen, N. Hanikel, M. I. Hossain, R. W. Flaig, X. Pei, A. Amin, M. D. Doherty, R. K. Impastato, T. G. Glover, D. R. Moore and O. M. Yaghi, *J. Am. Chem. Soc*. 2022, **144**, 2387–2396.

4 S.-B. He, A.-L. Hu, Q.-Q. Zhuang, H.-P. Peng, H.-H. Deng, W. Chen and G.-L. Hong, *ChemBioChem*, 2020, **21**, 978–984.

5 J. Chen, Q. Ma, M. Li, D. Chao, L. Huang, W. Wu, Y. Fang and S. Dong, *Nat. Commun*. 2021, **12**, 3375.

6 C. Gonçalves, R. M. Rodriguez-Jasso, N. Gomes, J. A. Teixeira and I. Belo, *Anal. Methods*, 2010, **2**, 2046–2048.

7 H. H. Gorris and D. R. Walt, *J. Am. Chem. Soc.* 2009, **131**, 6277–6282.

8 Y. Dong, Y. Chi, X. Lin, L. Zheng, L. Chen and G. Chen, *Phys. Chem. Chem. Phys.* 2011, **13**, 6319–6324.

9 K. P. Bhabak and G. Mugesh, *Chem. Eur. J.* 2007, **13**, 4594–4601.

10 A. V Peskin and C. C. Winterbourn, *Clin. Chim. Acta*, 2000, **293**, 157–166.

11 C. Creutz and N. Sutin, *Proc. Natl. Acad. Sci. U. S. A*. 1973, **70**, 1701–1703.

12 D. L. Mendez, S. E. Babbitt, J. D. King, J. D'Alessandro, M. B. Watson, R. E. Blankenship, L. M. Mirica and R. G. Kranz, *Proc. Natl. Acad. Sci. U. S. A*. 2017, **114**, 2235–2240.

13 X. Chen, X. Tian, I. Shin and J. Yoon, *Chem. Soc. Rev*. 2011, **40**, 4783–4804.

14 M. H. Groothaert, J. A. van Bokhoven, A. A. Battiston, B. M. Weckhuysen and R. A. Schoonheydt, *J. Am. Chem. Soc*. 2003, **125**, 7629–7640.

15 M. Sha, L. Rao, W. Xu, Y. Qin, R. Su, Y. Wu, Q. Fang, H. Wang, X. Cui, L. Zheng, W. Gu and C. Zhu, *Nano Lett*. 2023, **23**, 701–709.

16 Y. Li, X. Cai, S. Chen, H. Zhang, H. Zhang, J. Hong, B. H. Chen, D.-H. Kuo and W. Wang, *ChemSusChem*.

17 M. Li, J. Chen, W. Wu, Y. Fang and S. Dong, *J. Am. Chem. Soc*. 2020, **142**, 15569–15574.

18 Y. Sun, C. Zhao, N. Gao, J. Ren and X. Qu, *Chem. Eur. J.* 2017, **23**, 18146–18150.

19 Y. Liu, H. Wu, Y. Chong, W. G. Wamer, Q. Xia, L. Cai, Z. Nie, P. P. Fu and J.-J. Yin, *ACS Appl. Mater. Interfaces*, 2015, **7**, 19709–19717.
